# MIS12 Is Required for Kinetochore‐Microtubule Attachment in Oocyte Meiosis

**DOI:** 10.1002/advs.76171

**Published:** 2026-06-24

**Authors:** Jian Li, Chun‐Hui Zhang, Yong Wang, Cheng‐Yuan Li, Da‐Rong Wen, Xi Xia, Liang Zhou, Wei‐Ping Qian, Qing‐Yuan Sun, Chang‐Zhong Li

**Affiliations:** ^1^ Department of Reproductive Medicine Peking University Shenzhen Hospital Shenzhen China; ^2^ Fertility Preservation Lab Reproductive Medicine Center The Affiliated Guangdong Second Provincial General Hospital of Jinan University Guangzhou China

**Keywords:** K‐MT attachment, Meiosis, MIS12, Oocyte, TUBB3

## Abstract

Accurate kinetochore‐microtubule (K‐MT) attachment is crucial for chromosome segregation in oocyte meiosis. The NDC80 complex, a subcomplex of the outer kinetochore KNL1‑MIS12‑NDC80 (KMN) network, is canonically responsible for K‑MT attachment. Here, by employing a conditional knockout (cKO) mouse model, combined with protein localization analysis for MIS12 following chromosome spreading, we demonstrate that endogenous MIS12 localizes to kinetochores in both mouse and human oocytes, and depleting MIS12 severely disrupts K‐MT attachment in both meiotic divisions, independent of outer kinetochore protein NDC80. Crucially, we identified that MIS12 directly interacts with β‐tubulin (including TUBB3 and TUBB5), which is essential for microtubule attachment to kinetochores, revealing a previously unknown mechanism of K‐MT interaction. Furthermore, we definitively show that MIS12 is required for spindle assembly checkpoint (SAC) signaling by stabilizing the KNL1 assembly. Our findings not only resolve the previous controversy by establishing the canonical and essential role of kinetochore‐localized MIS12 in oocytes but also redefine its molecular function through its direct binding to microtubules, providing a new paradigm for KMN network‐mediated K‐MT attachment in mammalian oocyte meiosis.

## Graphical abstract

A model depicting the role of MIS12 in K‐MT attachment during oocyte meiosis. The presence of MIS12 stabilizes bipolar K‐MT attachments by maintaining the function of NDC80 and its interaction with TUBB. This, in turn, promotes KNL1 assembly and subsequent SAC protein recruitment to kinetochores. Conversely, the absence of MIS12 perturbs bipolar K‐MT attachment and abolishes the kinetochore localization of both KNL1 and SAC components.

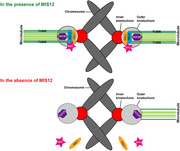

## 1 Introduction

Meiosis is a specialized cell division process essential for sexual reproduction, resulting in the formation of haploid gametes from diploid germ cells. In females, this process is particularly critical due to the susceptibility of oocytes to aneuploidy, which is a leading cause of congenital disabilities and reproductive failure [1, 2]. Chromosome segregation accuracy largely depends on the proper function of the kinetochore, a protein complex assembled at the centromere of chromosomes, and its interaction with spindle microtubules [3]. Therefore, correct kinetochore‐microtubule (K‐MT) attachment is a prerequisite for correct chromosome segregation.

The kinetochore comprises inner and outer layers. The inner layer, which anchors the structure to the centromere, consists of multiple CENP proteins organized into subcomplexes (e.g., CENP‐C, CENP‐LN, CENP‐HIKM, CENP‐OPQUR, and CENP‐TWSX) that collectively form the constitutive centromere‐associated network (CCAN). Assembled atop this foundation, the outer layer is primarily composed of the ten‐subunit KMN network—containing the NDC80, MIS12, and KNL1 complexes—which directly mediates microtubule attachment and recruits the spindle assembly checkpoint (SAC) machinery [3].

The NDC80 complex, a heterotetrameric assembly of NDC80, NUF2, SPC24, and SPC25, constitutes the essential microtubule receptor at the kinetochore [4, 5, 6]. More specifically, microtubule attachment is principally facilitated by the N‐terminal calponin homology (CH) domains within the NDC80‐NUF2 heterodimer [7, 8, 9, 10, 11]. A central feature of this interaction is the direct binding between the basic N‐terminal domain of the NDC80 subunit and the acidic E‐hook of both α‐ and β‐tubulin [10, 11, 12]. The role of the NUF2 CH domain is not to contact the microtubule directly, but to crucially stabilize the binding interface [8, 13]. The primary role of the KNL1 complex (composed of KNL1 and ZWINT) is to orchestrate the SAC signaling. This is achieved by recruiting essential SAC components, including Bub1, BubR1, and Bub3, via its multiple conserved motifs [14, 15]. The MIS12 complex (composed of MIS12, DSN1, NSL1, and PMF1) functions as a critical interaction hub that links the KMN network to the inner kinetochore by binding to both CENP‐C and CENP‐T [16, 17]. Additionally, it provides binding sites for KNL1 and the NDC80 complex [18, 19, 20]. Depletion of MIS12 complex subunits in human or chicken cells results in mitotic delay, accompanied by chromosome misalignment and impaired biorientation, as well as reduced centromere stretch and weaker kinetochore microtubule bundles of aligned chromosomes [21], indicating that the MIS12 complex is essential for mitotic kinetochore assembly and accurate chromosome segregation in vertebrates. Although the MIS12 complex itself does not bind to microtubules, it potentiates the microtubule‐binding affinity of the NDC80 complex during mitosis [22]. A latest research further reveals that dynamic phosphorylation of the MIS12 complex regulates the expansion‐compaction transition of the kinetochore outermost layer, a critical mechanism for ensuring proper K‐MT attachment and faithful chromosome segregation [23].

Interestingly, recent studies by us and others have revealed that members of the KMN network play noncanonical roles in regulating the stability of cyclin B (including cyclin B1 and B2) during meiotic maturation of oocytes [24, 25, 26, 27]. Of particular interest, one study reported that MIS12 localizes to spindle poles rather than kinetochores in mouse oocytes [26]. Moreover, knockdown of MIS12 did not impair K‐MT attachment during oocyte meiosis [26]. Due to technical limitations, the exact role of MIS12 in K‐MT attachment during oocyte meiosis remains unclear.

In the present study, we established a conditional knockout (cKO) mouse model to investigate the function of MIS12 during oocyte meiotic maturation. We found that MIS12 is required for proper K‐MT attachment in both meiosis I and meiosis II. The K‐MT attachment defects observed in *Mis12*‐deleted oocytes were similar to those seen in *Ndc80*‐deficient oocytes [28]. Notably, NDC80 localization at kinetochores was maintained in the absence of MIS12. Importantly, we demonstrated that MIS12 is in fact localized to kinetochores in mouse and human oocytes, and that its direct interaction with β‐tubulin (including TUBB3 and TUBB5) is essential for K‐MT attachment. In addition, MIS12 was shown to be necessary for SAC function by regulating the stability of the KNL1 assembly. Together, these findings uncover a previously unrecognized role for MIS12 in K‐MT attachment during oocyte meiosis, providing new insights into the function of the KMN network in regulating microtubule attachment in oocytes.

## 2 Results

### 2.1 Mis12 Deletion Disrupts Spindle and Chromosome Dynamics in Metaphase II Oocytes

**FIGURE 1:**
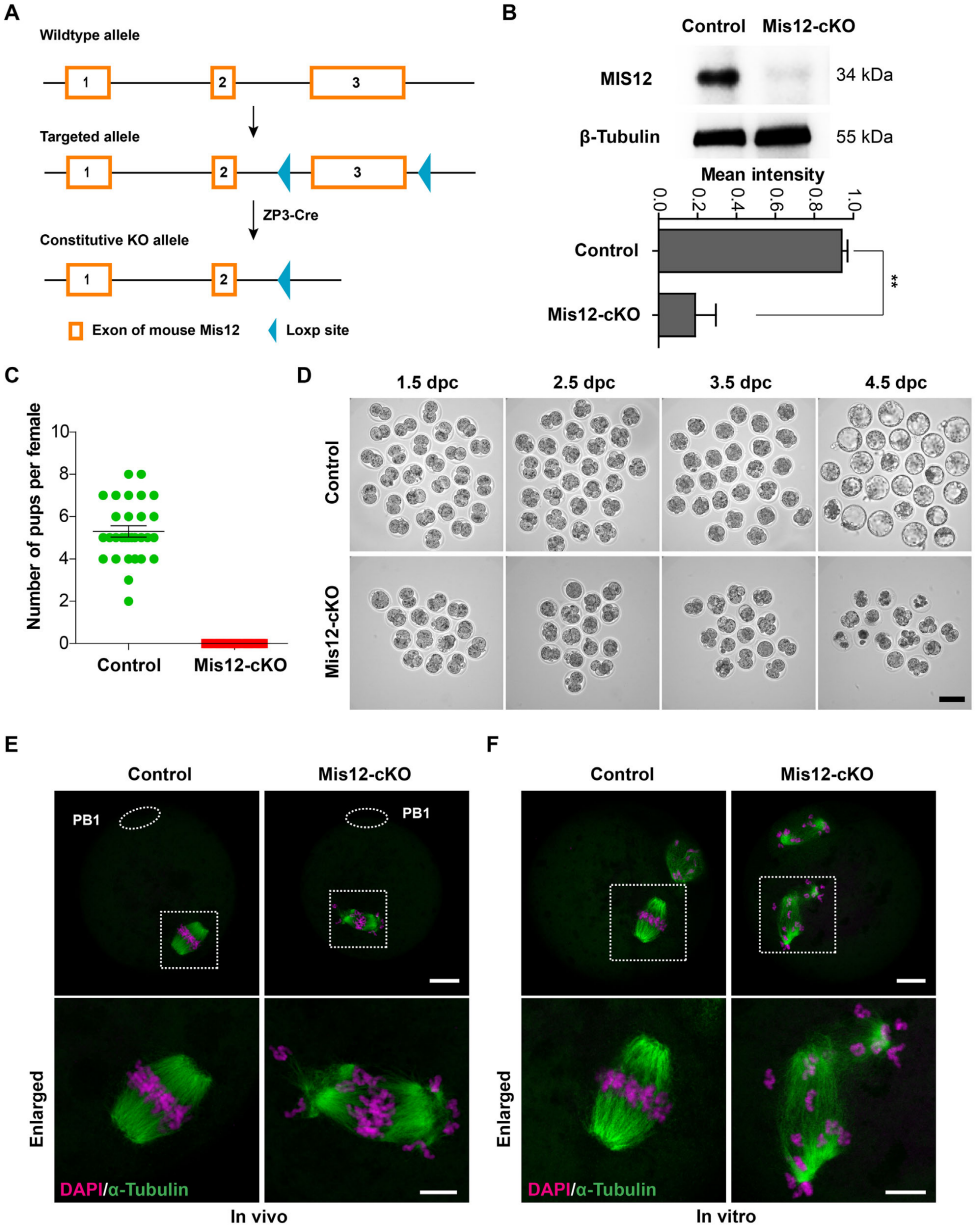
*Mis12* knockout causes female infertility by disrupting spindle and chromosome dynamics in meiosis II of oocyte. (A) Strategy for generating the *Mis12* conditional knockout (cKO) allele. The wild‐type allele (top) was modified by inserting LoxP sites (blue triangles) flanking exon 3 (orange). Cre‐mediated recombination under the ZP3 promoter deletes exon 3, producing the cKO allele (bottom). (B) Western blot confirms efficient MIS12 depletion in *Mis12*‐deleted oocytes. β‐Tubulin serves as a loading control. Each lane was loaded with 150 oocytes. Western blot quantification was analyzed, and the difference was statistically significant (Data are presented as mean ± SEM; ^**^ *p* < 0.05). Three independent experiments were conducted. (C) MIS12 deletion causes female infertility. The number of pups per female is significantly reduced in cKO mice (red) (n = 30) compared to controls (green) (n = 30). Each dot represents one litter of offspring mice. (D) Embryos from *Mis12*‐cKO females exhibit developmental failure. No blastocysts were observed in the *Mis12*‑cKO group (n = 43), whereas blastocysts were observed in the control group (n = 39). Representative images at various days post coitum (dpc) are shown. Scale bar: 100 µm. Three independent experiments were conducted. (E, F) Chromosome alignment and spindle assembly are disrupted in *Mis12*‐deleted metaphase II oocytes after either (E) in vivo (control, n = 21; *Mis12*‐cKO, n = 27) or (F) in vitro (control, n = 25; *Mis12*‐cKO, n = 26) maturation. Oocytes were stained for α‐tubulin (green, microtubules) and DNA (magenta, chromosomes). The first polar body (PB1) is indicated by a dotted ellipse in (E). Scale bars: 20 µm (overview), 10 µm (enlarged view). Three independent experiments were conducted.

To investigate the role of MIS12 in oocyte meiotic maturation, we generated a conditional knockout (cKO) mouse model using ZP3‐Cre recombinase (Figure 1A). Western blot analysis confirmed a marked reduction of MIS12 protein in *Mis12*‐deleted oocytes compared with controls (Figure 1B). Fertility assessments over at least six months revealed complete female sterility in *Mis12*‐cKO mice (Figure 1C). To determine the cause of infertility, we examined embryonic development following in vivo fertilization. Although *Mis12*‐deleted zygotes reached the two‐cell stage, their development arrested thereafter, and embryos degenerated by 4.5 days post coitum (dpc), whereas control embryos developed to the blastocyst stage (Figure 1D). This suggests that *Mis12*‐deleted oocytes are defective and likely incapable of supporting early embryo development.

To assess the state of mature oocytes lacking MIS12, we collected metaphase II (MII) oocytes from the oviductal ampulla of control and *Mis12*‐cKO females after PMSG and hCG stimulation and performed immunofluorescence staining. As expected, control MII oocytes exhibited well‐aligned chromosomes at the metaphase plate of bipolar spindles. In contrast, *Mis12*‐deleted oocytes displayed severe meiotic disruption: chromosomes were scattered and misaligned, with numerous unattached chromosomes near the spindle poles, and microtubule organization was grossly aberrant (Figure 1E). A similar phenotype was observed in in vitro‐matured MII oocytes (Figure 1F). These results indicate that MIS12 is essential for K‐MT attachment during oocyte meiosis.

### 2.2 MIS12 Is Required for K‐MT Attachment in Oocyte Meiosis I

**FIGURE 2:**
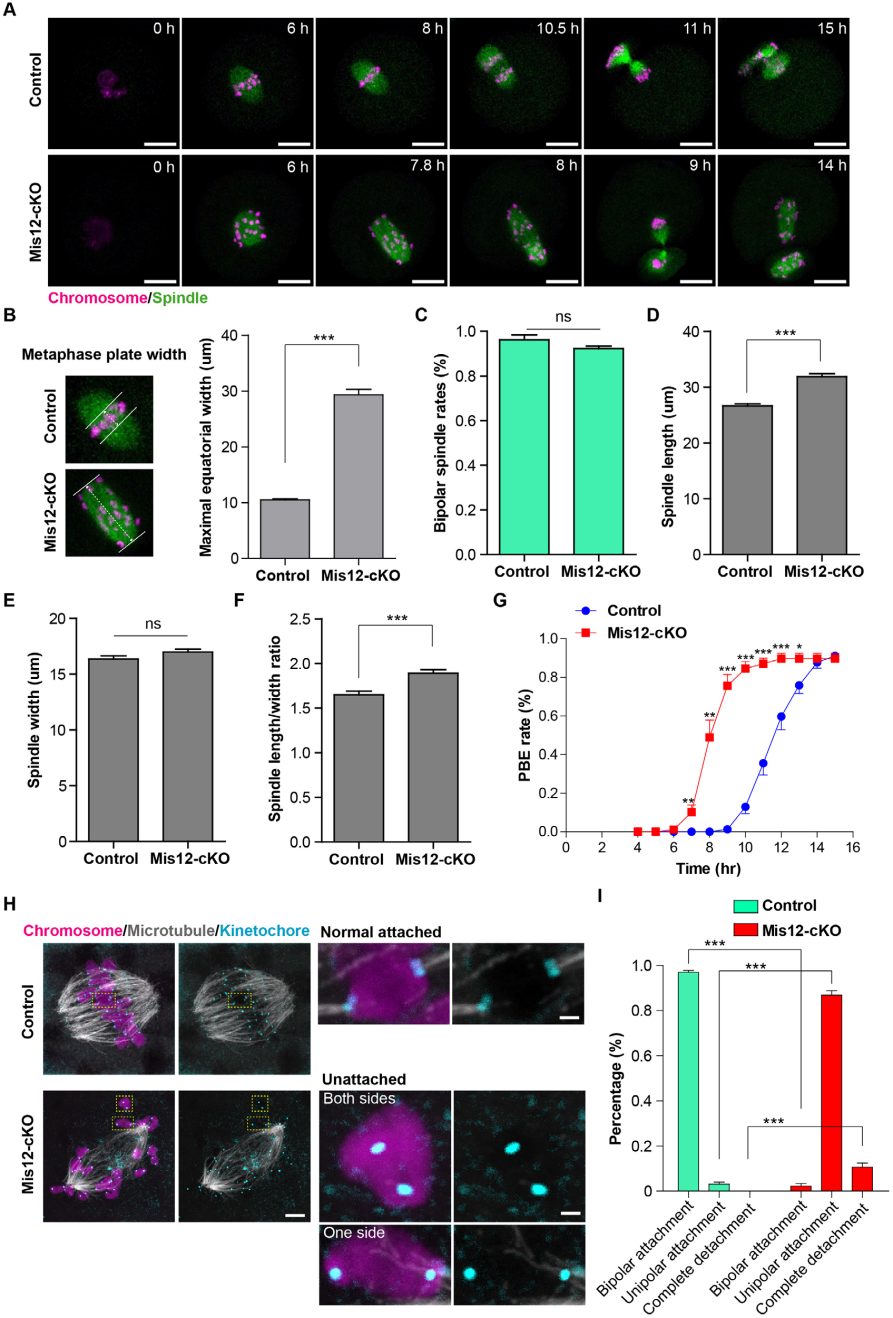
MIS12 is required for K‐MT attachment in mouse oocytes. (A) Representative time‐lapse images of control and *Mis12*‐deleted oocytes expressing EGFP‐MAP4 (microtubules, green) and H2B‐mCherry (chromosomes, magenta), captured over 15 h with a frame interval of 10 min. Scale bar: 20 µm. Three independent experiments were conducted. (B) MIS12 deletion disrupts chromosome alignment. Quantification of the maximal equatorial chromosome width in meiosis I shows significant widening in mutant oocytes (n = 23) compared with control oocytes (n = 30). Data are presented as mean ± SEM; ^***^ *p* < 0.0001. (C) Analysis of spindle bipolarity at metaphase I in control (n = 47) and *Mis12*‐cKO (n = 63) mouse oocytes. Data are presented as mean ± SEM; ns, not significant. (D) Analysis of spindle length at metaphase I in control (n = 39) and *Mis12*‐cKO (n = 52) mouse oocytes. Data are presented as mean ± SEM; ^***^ *p* < 0.0001. (E) Analysis of spindle width at metaphase I in control (n = 39) and *Mis12*‐cKO (n = 52) mouse oocytes. Data are presented as mean ± SEM; ns, not significant. (F) Analysis of the spindle length/width ratio at metaphase I in control (n = 39) and *Mis12*‐cKO (n = 52) mouse oocytes. Data are presented as mean ± SEM; ^***^ *p* < 0.0001. (G) *Mis12* deletion accelerates meiosis I progression (control, n = 138; *Mis12*‐cKO, n = 152). Polar body extrusion (PBE) rates were recorded hourly from 4 to 15 h. Data are presented as mean ± SEM; ^***^p < 0.0001, ^**^ *p* < 0.001, ^*^ *p* < 0.01. (H) MIS12 is essential for stable K‐MT attachment in meiosis I. Cold‐stable assay reveals increased microtubule detachment in *Mis12*‐deleted oocytes (n = 16) compared with control oocytes (n = 13). Oocytes were immunostained for α‐tubulin (white), ACA (kinetochores, blue), and DNA (magenta). Scale bars: 5 µm (overview), 1 µm (enlarged view). Three independent experiments were conducted. (I) Quantification of chromosome attachment errors from (H). The graph depicts the percentage of oocyte bivalents exhibiting normal bipolar attachment, unipolar (single‐side) attachment, or complete (double‐side) detachment. Data are presented as mean ± SEM; ^***^ *p* < 0.0001.

Given the disrupted spindle and K‐MT attachment in Mis12‐cKO MII oocytes, we asked whether MIS12 is similarly required in meiosis I. Using live‐cell confocal imaging of oocytes expressing MAP4‐EGFP (to label microtubules) and H2B‐mCherry (to label chromosomes), we monitored spindle and chromosome dynamics throughout meiotic progression. In control oocytes, bipolar spindle assembly and chromosome alignment proceeded normally (Figure 2A,B and Video S1). In *Mis12*‐deleted oocytes, the bipolar spindle could be established during meiosis I; however, chromosomes failed to congress and were randomly distributed. Surprisingly, despite this disorganization, chromosomes still segregated and the first polar body was extruded in *Mis12*‐deleted oocytes (Figure 2A,B and Video S2). Although the spindle bipolarity was not significantly affected (Figure 2C), the metaphase I spindle became much longer in Mis12‐deleted oocytes compared with control oocytes (Figure 2D–F), likely resulting from an imbalance in K‐MT attachment. Meiotic progression was significantly accelerated in *Mis12*‐deleted oocytes, with polar body extrusion (PBE) occurring at least 2–3 h earlier than in controls (Figure 2G), suggesting possible SAC impairment. Consequently, the aneuploidy rate was expected to be significantly higher in *Mis12*‐deleted MII oocytes (Figure S1).

To evaluate K‐MT attachment status, we stained kinetochores and microtubules in cold‐treated metaphase I oocytes. Whereas control oocytes exhibited robust bipolar K‐MT attachments, *Mis12*‐deleted oocytes showed severe attachment defects: a majority of bivalents (86.88% ± 1.93%, vs. 3.08% ± 0.90% in controls) were attached by microtubules from only one pole and pulled poleward, and nearly 10% (10.63% ± 1.88%, vs. 0.07% ± 0.07% in controls) remained entirely unattached (Figure 2H,I).

Together, these data demonstrate that MIS12 is essential for K‐MT attachment in both meiotic divisions. In its absence, K‐MT attachment is severely compromised, leading to aberrant chromosome segregation and premature meiotic progression, likely due to SAC dysfunction. These findings underscore the critical role of MIS12 in maintaining chromosomal stability during oocyte meiosis.

### 2.3 NDC80 Is Still Localized on the Kinetochore in the Mis12‐Deleted Oocytes

**FIGURE 3:**
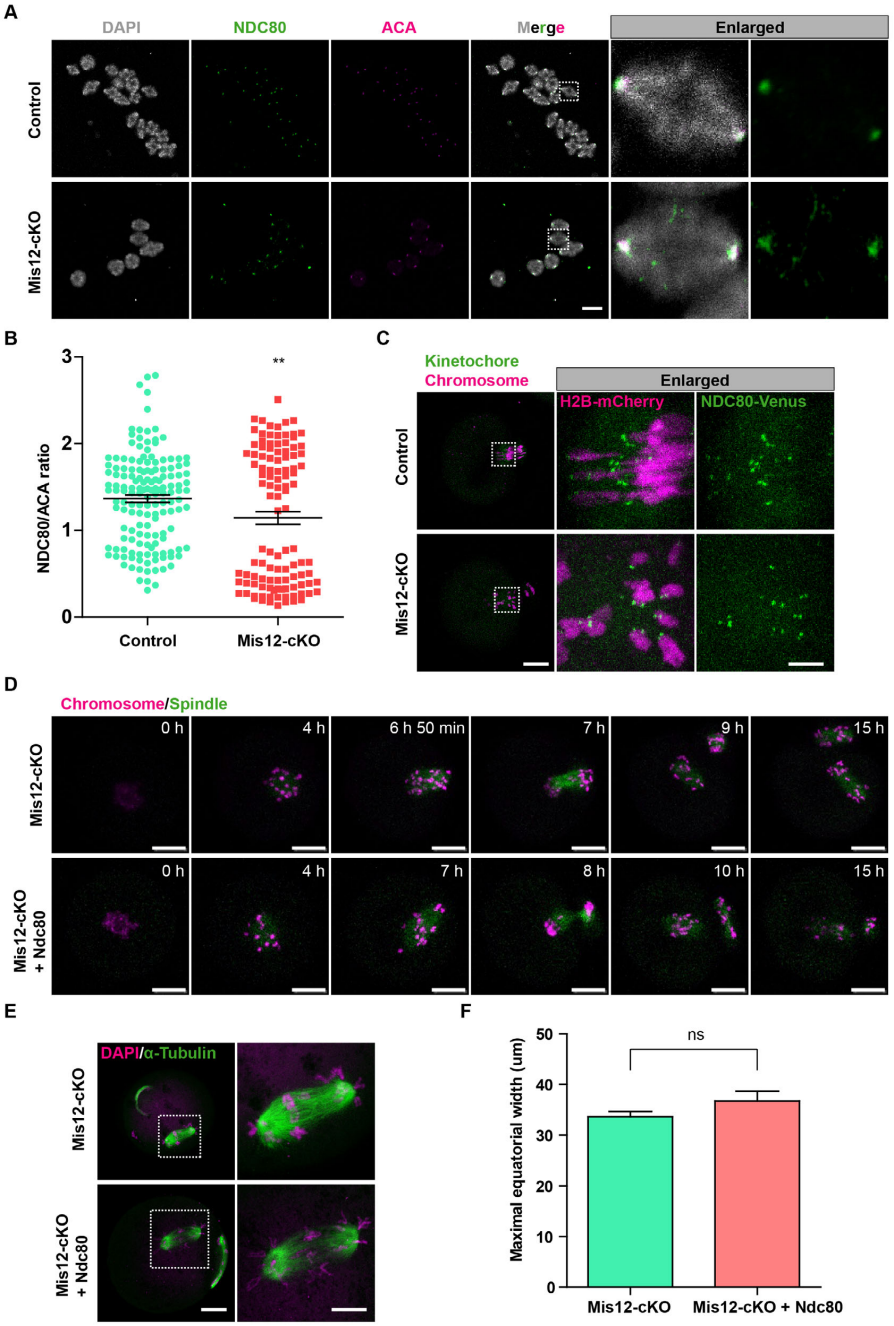
NDC80 is retained at kinetochores in *Mis12*‐deleted oocytes. (A) Endogenous NDC80 localizes to kinetochores in the absence of MIS12. Control and *Mis12*‐deleted metaphase I oocytes were immunostained with antibodies against NDC80 (green) and centromeres (ACA, magenta). DNA was counterstained with DAPI (grayscale). Scale bars: 10 µm (overview) and 5 µm (enlarged view). Three independent experiments were conducted. (B) Quantification of the relative kinetochore levels of NDC80 in control (n = 152) and *Mis12*‐deleted (n = 110) oocytes. The fluorescence intensity ratio of NDC80 to ACA at individual kinetochores is shown for each genotype. Data are presented as mean ± SEM; ^**^ *p* < 0.05. The number of kinetochores (n) analyzed is indicated. (C) Exogenous NDC80 correctly targets to kinetochores in both *Mis12*‐deleted oocytes (n = 11) and control oocytes (n = 10). Live oocytes microinjected with mRNA encoding NDC80‐Venus (green) and H2B‐mCherry (magenta) were imaged by confocal microscopy. Scale bars: 20 µm (overview) and 5 µm (enlarged view). (D) Representative time‐lapse images of *Mis12*‐deleted oocytes (n = 15) and *Mis12*‐deleted oocytes with *Ndc80* supplementation (n = 12) expressing EGFP‐MAP4 (microtubules, green) and H2B‐mCherry (chromosomes, magenta), captured over 15 h with a frame interval of 10 min. Scale bar: 20 µm. Three independent experiments were conducted. (E) Immunofluorescence analysis of chromosome and spindle morphologies of MII oocytes in *Mis12*‐deleted oocytes (n = 15) and *Mis12*‐deleted oocytes with *Ndc80* supplementation (n = 12). Oocytes were stained for α‐tubulin (green, microtubules) and DNA (magenta, chromosomes). Scale bars: 20 µm (overview), 10 µm (enlarged view). (F) *Ndc80* supplementation failed to rescue chromosome alignment defects in *Mis12*‐deleted oocytes. Data are presented as mean ± SEM; ns, not significant.

The NDC80 protein, situated at the outermost periphery of the kinetochore, acts as an essential linker between kinetochores and microtubules. Deletion of Ndc80 results in chromosome alignment and K‐MT attachment defects [28], similar to those seen in *Mis12*‐deleted oocytes. This suggests that NDC80 may be absent in *Mis12*‐deleted oocytes. Immunofluorescence staining after chromosome spread was performed to visualize the presence of endogenous NDC80 in control and *Mis12*‐deleted oocytes (Figure 3A). Despite a reduction in its expression level upon *Mis12* deletion, NDC80 remained robustly localized at the kinetochore (Figure 3A,B), indicating that its kinetochore localization is largely independent of MIS12 function. Additionally, using an exogenous NDC80‐Venus fusion construct, it was observed that NDC80‐Venus was clearly detected at the kinetochores in *Mis12*‐deleted oocytes (Figure 3C). Actually, unlike *Ndc80*‐deficient oocytes, which fail to assemble a bipolar spindle in meiosis I [28], oocytes lacking MIS12 form bipolar spindles (Figure 2A–C). This phenotypic difference strongly implies that NDC80 retains its function in the absence of MIS12. In conclusion, although MIS12 appears to be essential for the proper assembly and function of the kinetochore, its depletion does not severely impair NDC80 localization and function. To test this better, we microinjected exogenous *Ndc80* mRNA into *Mis12*‐deleted oocytes in an attempt to rescue K‐MT attachment defects. As expected, both live‐cell imaging (Figure 3D and Videos S3 and S4) and immunofluorescence (Figure 3E,F) revealed that exogenous NDC80 did not improve chromosome alignment in meiosis I or II, demonstrating that K‐MT attachment remained compromised.

Given that NDC80 persists at kinetochores in the absence of MIS12, yet fails to rescue upon supplementation, alternative mechanisms by which MIS12 contributes to K‐MT attachment must exist. One compelling possibility is that MIS12 directly regulates microtubule binding.

### 2.4 MIS12 Regulates K‐MT Attachment Independent of NUF2 in Oocytes

**FIGURE 4:**
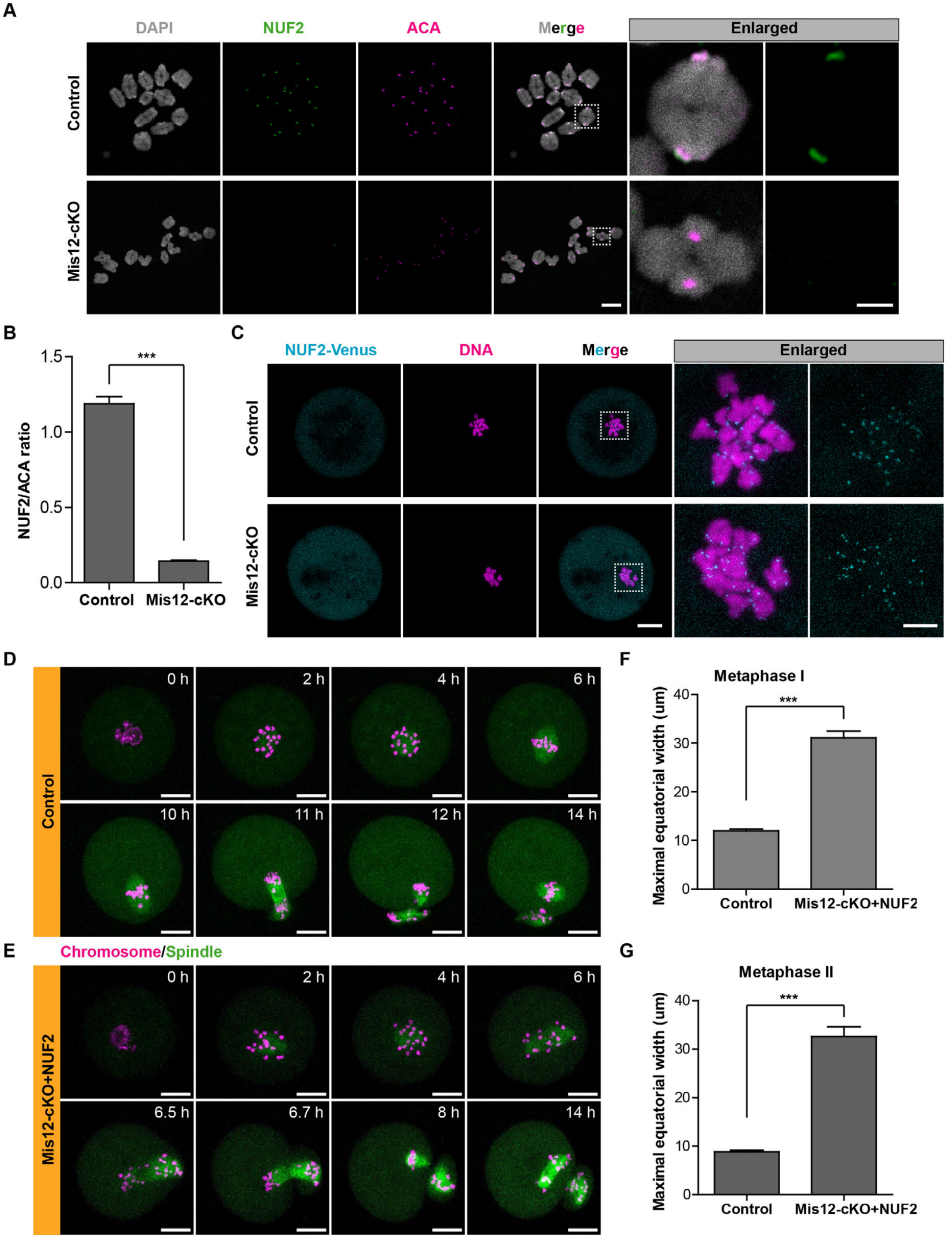
Exogenous NUF2 fails to rescue K‐MT attachment in *Mis12*‐deleted oocytes. (A) Kinetochore localization of NUF2 is lost upon *Mis12* deletion. Oocytes were immunostained with anti‐NUF2 and anti‐ACA antibodies, DNA is counterstained with DAPI. Scale bars: 10 µm (overview) and 5 µm (enlarged view). Three independent experiments were conducted. (B) Quantification of the NUF2/ACA ratio from (A) in control (n = 99) and *Mis12*‐deleted (n = 100) oocytes. Data are presented as mean ± SEM; ^***^ *p* < 0.0001. The number of kinetochores analyzed (n) is indicated. (C) Exogenously expressed NUF2‐Venus localizes correctly to kinetochores in both control (n = 8) and *Mis12*‐deleted (n = 10) oocytes. Scale bars: 20 µm (overview) and 5 µm (enlarged view). (D, E) Representative time‐lapse confocal images of (D) control oocytes (n = 11) and (E) *Mis12*‐deleted oocytes microinjected with *Nuf2* mRNA (n = 12), both expressing EGFP‐MAP4 (microtubules, green) and H2B‐mCherry (chromosomes, magenta), captured over 15 h with a frame interval of 10 min. Scale bar: 20 µm. Twice independent experiments were conducted. (F, G) Chromosome alignment is not rescued by exogenous NUF2 in (F) meiosis I or (G) meiosis II in *Mis12*‐deleted oocytes. Data are presented as mean ± SEM; ^***^ *p*<0.0001.

To further explore the impact of MIS12 deficiency on kinetochore function, we examined the expression of another critical kinetochore protein, NUF2, which is also necessary for K‐MT attachment [13]. The results showed a clear loss in NUF2 localization and expression on kinetochores in *Mis12*‐deleted oocytes (Figure 4A,B). Given that NUF2 is essential for building a complete NDC80 subcomplex and is crucial for maintaining the stability of the outer kinetochore surface for connection to microtubules, the loss of NUF2 further confirmed the vital role of MIS12 in maintaining kinetochore integrity and functionality. This raises the question of whether the phenotype of *Mis12*‐deleted oocytes is a result of the MIS12‐dependent loss of NUF2. To address this point, we attempted to rescue the abnormal phenotype of *Mis12*‐deleted oocytes by supplementing exogenous NUF2. Firstly, we examined the expression and localization of exogenous NUF2 tagged with Venus fluorescent protein in the control and *Mis12*‐deleted oocytes. It was shown that NUF2‐Venus was well expressed and localized at the kinetochores in both groups of oocytes (Figure 4C). Then we inspected the spindle assembly and chromosome separation during meiotic progression by live imaging in control and *Mis12*‐deleted oocytes after NUF2 supplementation. We found that this remedial measure was unable to effectively improve the chromosome separation anomalies (Figure 4D,E and Videos S5 and S6). Neither in meiosis I nor in meiosis II were the chromosome alignments rescued (Figure 4F,G). These results indicate that simply replenishing NUF2 cannot substitute for the function of MIS12, further suggesting that MIS12 might influence K‐MT connections through other mechanisms.

In this regard, the significance of MIS12 in kinetochore structure and function is highlighted by these findings. Even though NUF2 is important, MIS12 appears to be essential for K‐MT attachment regardless of NUF2.

### 2.5 MIS12 Localizes at Kinetochores in Mouse and Human Oocytes

**FIGURE 5:**
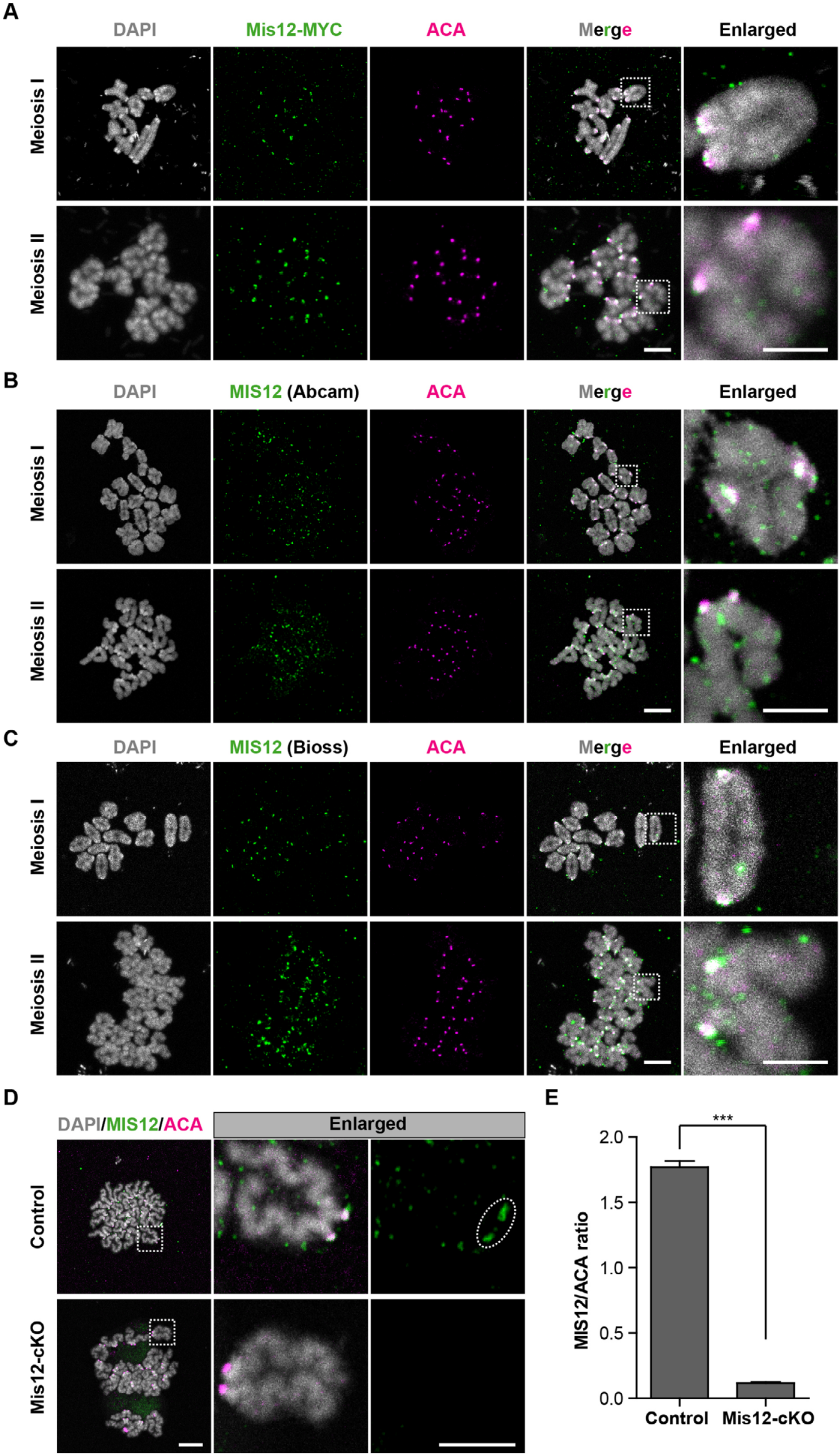
MIS12 localizes to kinetochores in mouse oocytes. (A) Exogenous MIS12 robustly localizes to kinetochores during both meiosis I (n = 13) and meiosis II (n = 12). Oocytes were immunostained with anti‐MYC and anti‐ACA antibodies, DNA is counterstained with DAPI. Three independent experiments were conducted. (B) Endogenous MIS12 clearly localizes to kinetochores in meiosis I (n = 15) and meiosis II (n = 13), as validated using antibody from Abcam. Three independent experiments were conducted. (C) Endogenous MIS12 clearly localizes to kinetochores in meiosis I (n = 11) and meiosis II (n = 14), as validated using antibody from Bioss. Three independent experiments were conducted. (D) MIS12 signal is absent from kinetochores in *Mis12*‐deleted oocytes. Oocytes after spreading were immunostained with anti‐MIS12 (Abcam) and anti‐ACA antibodies. DNA is counterstained with DAPI (Applies to A‐D). Scale bars: 10 µm (overview), 5 µm (enlarged view) (Applies to A–D). Three independent experiments were conducted. Three independent experiments were conducted. (E) Quantification of the MIS12/ACA ratio from (D) in control (n = 145) and *Mis12*‐deleted (n = 148) oocytes. Data are presented as mean ± SEM; ^***^ *p* < 0.0001. The number of kinetochores analyzed (n) is indicated.

The localization of a protein is typically linked to its function, so it is probable that the MIS12 protein is situated on the kinetochores in oocytes due to the K‐MT attachment phenotype in *Mis12*‐deleted oocytes. However, a previous study reported that the MIS12 protein was only found at the spindle poles in mouse oocytes [26]. To clarify the localization of MIS12 in the oocyte, chromosome spreading was employed in the following examinations. First, the MIS12‐MYC fusion probe was utilized for analysis with an anti‐MYC antibody, circumventing the issue with the MIS12 antibody quality. The results revealed the clear presence of MIS12‐MYC at the kinetochores of both homologous chromosomes in meiosis I and sister chromatids in meiosis II (Figure 5A). Additionally, a Venus tag attached to either the C‐terminus or N‐terminus of MIS12 was also used to verify the kinetochore localization of MIS12 in mouse oocytes (Figure S2A,B). Then we attempted to analyze the localization of the endogenous MIS12 protein by utilizing antibodies from various manufacturers. As expected, the MIS12 protein was detected at the kinetochores during both meiosis I and meiosis II (Figure 5B,C). These data demonstrate that the MIS12 protein localizes at the kinetochores in mouse oocytes, and this localization mode well explains the K‐MT attachment phenotype in *Mis12*‐deleted oocytes. Additionally, we confirmed the lack of MIS12 protein on the kinetochores in *Mis12*‐deleted mouse oocytes (Figure 5D,E). This absence further supports the critical role of MIS12 in ensuring proper kinetochore function and chromosome segregation during meiosis. Moreover, the localization of endogenous MIS12 on kinetochores in human oocytes was confirmed by the same method (Figure S2C). These observations suggest a conserved function of MIS12 across species, highlighting its essential role in meiotic maturation. The consistent presence of MIS12 at kinetochores implies that it may be involved in the assembly and stability of the K‐MT attachments necessary for accurate chromosome segregation in oocytes.

### 2.6 Artificially Tethering MIS12 to the NDC80 Can Rescue the Phenotype of Mis12‐Deleted Oocytes

**FIGURE 6:**
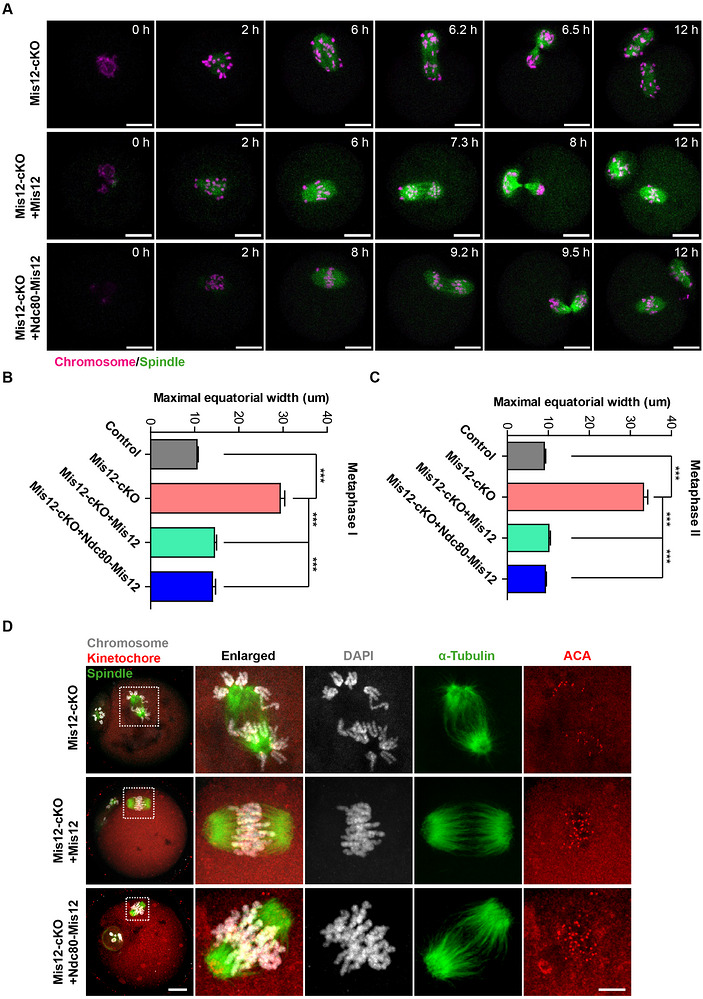
NDC80‐anchoring MIS12 rescues K‐MT attachment in *Mis12*‐deleted oocytes. (A) Representative time‐lapse images of oocytes expressing EGFP‐MAP4 (microtubules) and H2B‐mCherry (chromosomes), comparing *Mis12*‐deleted oocytes with or without supplementation of *Mis12* mRNA or *Ndc80*‐*Mis12* fusion mRNA. Images were captured over 13 h with a frame interval of 10 min. Scale bar: 20 µm. Three independent experiments were conducted. (B) Chromosome alignment in meiosis I is restored by expressing either MIS12 or the NDC80‐MIS12 fusion protein in *Mis12*‐deleted oocytes (Control, n = 18; *Mis12*‐cKO, n = 19; *Mis12*‐cKO + *Mis12*, n = 19; *Mis12*‐cKO + *Ndc80*‐*Mis12*, n = 21). Data are presented as mean ± SEM; ^***^ *p*<0.0001. (C) Chromosome alignment in meiosis II is restored by expressing either MIS12 or the NDC80‐MIS12 fusion protein in *Mis12*‐deleted oocytes (Control, n = 17; *Mis12*‐cKO, n = 18; *Mis12*‐cKO + *Mis12*, n = 16; *Mis12*‐cKO + *Ndc80*‐*Mis12*, n = 19). Data are presented as mean ± SEM; ^***^ *p*<0.0001. (D) K‐MT attachment in meiosis II is successfully rescued by MIS12 or NDC80‐MIS12 (*Mis12*‐cKO, n = 19; *Mis12*‐cKO + *Mis12*, n = 16; *Mis12*‐cKO + *Ndc80*‐*Mis12*, n = 17). Oocytes were immunostained with anti‐α‐tubulin and anti‐ACA antibodies, DNA is counterstained with DAPI. Scale bars: 20 µm (overview), 5 µm (enlarged view).

To define the essential role of MIS12 in K‐MT attachment, we performed a rescue experiment by reintroducing *Mis12* mRNA into *Mis12*‐deleted oocytes. Live imaging observation demonstrated that supplementing wild‐type *Mis12* effectively restored chromosome alignment in *Mis12*‐deleted oocytes during meiotic maturation (Figure 6A and Videos S7 and S8), indicating that it was the disruption of MIS12 that led to impaired K‐MT attachment. The improvement in chromosome alignment was significant in both meiosis I and meiosis II (Figure 6B,C), suggesting that K‐MT attachment was rescued by MIS12 supplementation. The results of the microtubule and kinetochore staining experiment further confirmed the successful rescue of K‐MT attachment by MIS12 in meiosis II (Figure 6D). To explore MIS12's role in K‐MT attachment better, we attempted to position MIS12 behind NDC80 on the outer kinetochore layer. A *Ndc80*‐*Mis12*‐*Venus* fusion vector was constructed to confirm its expression on kinetochores, and the *Ndc80‐Mis12* fused construct was used to rescue the phenotype of *Mis12*‐deleted oocytes. The results demonstrated that NDC80‐MIS12‐Venus was specifically localized at the kinetochores (Figure S3), and NDC80‐MIS12 successfully rescued the failure of chromosome alignment and K‐MT attachments in *Mis12*‐deleted oocytes (Figure 6A–D; and Videos S7 and S9). This discovery further highlights the essential role of MIS12 in maintaining correct kinetochore function during oocyte meiosis.

### 2.7 MIS12 Interacts With Microtubules by Binding to TUBB

**FIGURE 7:**
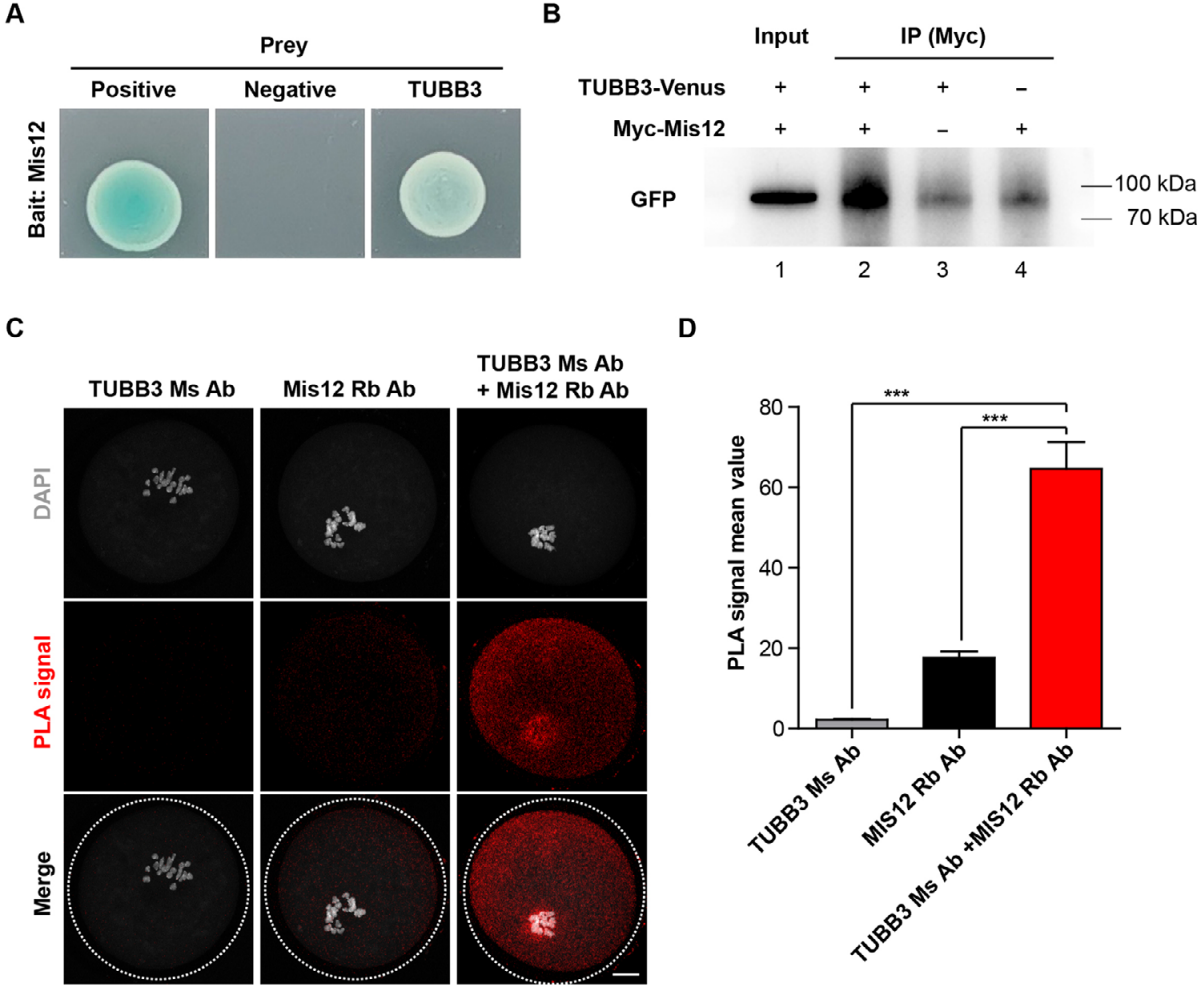
MIS12 interacts with TUBB3 in mouse oocytes. (A) Yeast two‐hybrid assay demonstrates an interaction between MIS12 (bait) and TUBB3 (prey, clone #36) using mouse ovary cDNA library. (B) Co‐immunoprecipitation (Co‐IP) validating the MIS12‐TUBB3 interaction. Lysates from 293T cells co‐expressing MYC‐MIS12 and TUBB3‐Venus (lane 2) were subjected to IP with an anti‐MYC antibody, followed by immunoblotting (IB) with an anti‐GFP antibody. Controls include cells expressing MYC‐MIS12 (lane 3) or TUBB3‐Venus (lane 4) alone. Input represents the total protein from co‐expressing cells (lane 1). Three independent experiments were conducted. (C) Proximity ligation assay (PLA) detecting endogenous MIS12‐TUBB3 interaction in oocytes. Wild‐type oocytes were co‐incubated with anti‐MIS12 (rabbit polyclonal) and anti‐TUBB3 (mouse monoclonal) antibodies (n = 17). Oocytes incubated with anti‐TUBB3 antibody (n = 14) or anti‐MIS12 antibody (n = 15) served as negative controls. DNA was counterstained with DAPI. Scale bar: 20 µm. Three independent experiments were conducted. (D) Quantification of PLA signals from (C). Data are presented as mean ± SEM; ^***^ *p* < 0.0001.

Now that MIS12 is essential for K‐MT attachment, which is independent of the NDC80, it is possible that MIS12 may interact directly with microtubules. Currently, the widely accepted view is that the NDC80 directly binds to microtubules and recruits microtubule‐binding proteins [29, 30]. Based on the present results, we were led to hypothesize that MIS12 could additionally interact with microtubules. To investigate this, a yeast two‐hybrid screen was employed, using *Mis12* as bait against a cDNA library derived from mouse ovaries. As predicted, the screen detected the presence of TUBB3 (tubulin, beta 3 class III), a member of the beta‐tubulin family (Figure 7A), indicating that MIS12 can interact with microtubules by binding to TUBB3. To verify this interaction, we created *Venus*‐tagged *Tubb3* and *Myc*‐tagged *Mis12* plasmids and transfected them into 293T cells to perform co‐immunoprecipitation (Co‐IP). Consistently, the results of the Co‐IP experiment further demonstrated that MIS12 can indeed interact with TUBB3 (Figure 7B). To make it clearer, we continued to examine this interaction in mouse oocytes by proximity ligation assay (PLA) analysis. A striking PLA signal, particularly around chromosomes, was observed in oocytes co‐incubated with anti‐TUBB3 and anti‐MIS12 antibodies (Figure 7C,D). This signal was barely detectable in control oocytes incubated with either antibody alone (Figure 7C,D). The results clearly show that MIS12 directly interacts with TUBB3 in mouse oocytes, indicating that MIS12 is a direct microtubule‐interacting protein at the outer kinetochore in mouse oocytes. Given that β‐tubulin comprises multiple isoforms, we next investigated whether MIS12 could also interact with other β‐tubulin family members. To this end, we performed additional PLA assays to examine the interaction between MIS12 and TUBB5, another major β‐tubulin isoform expressed in mouse oocytes. Intriguingly, our results confirmed that MIS12 also interacts with TUBB5 (Figure S4). These findings suggest that the MIS12–TUBB interaction is likely a general phenomenon and plays an essential role in K‐MT attachment in mouse oocytes.

### 2.8 MIS12 Tethering on the Chromosomes Can Rescue the Phenotype of Mis12‐Deleted Oocytes

**FIGURE 8:**
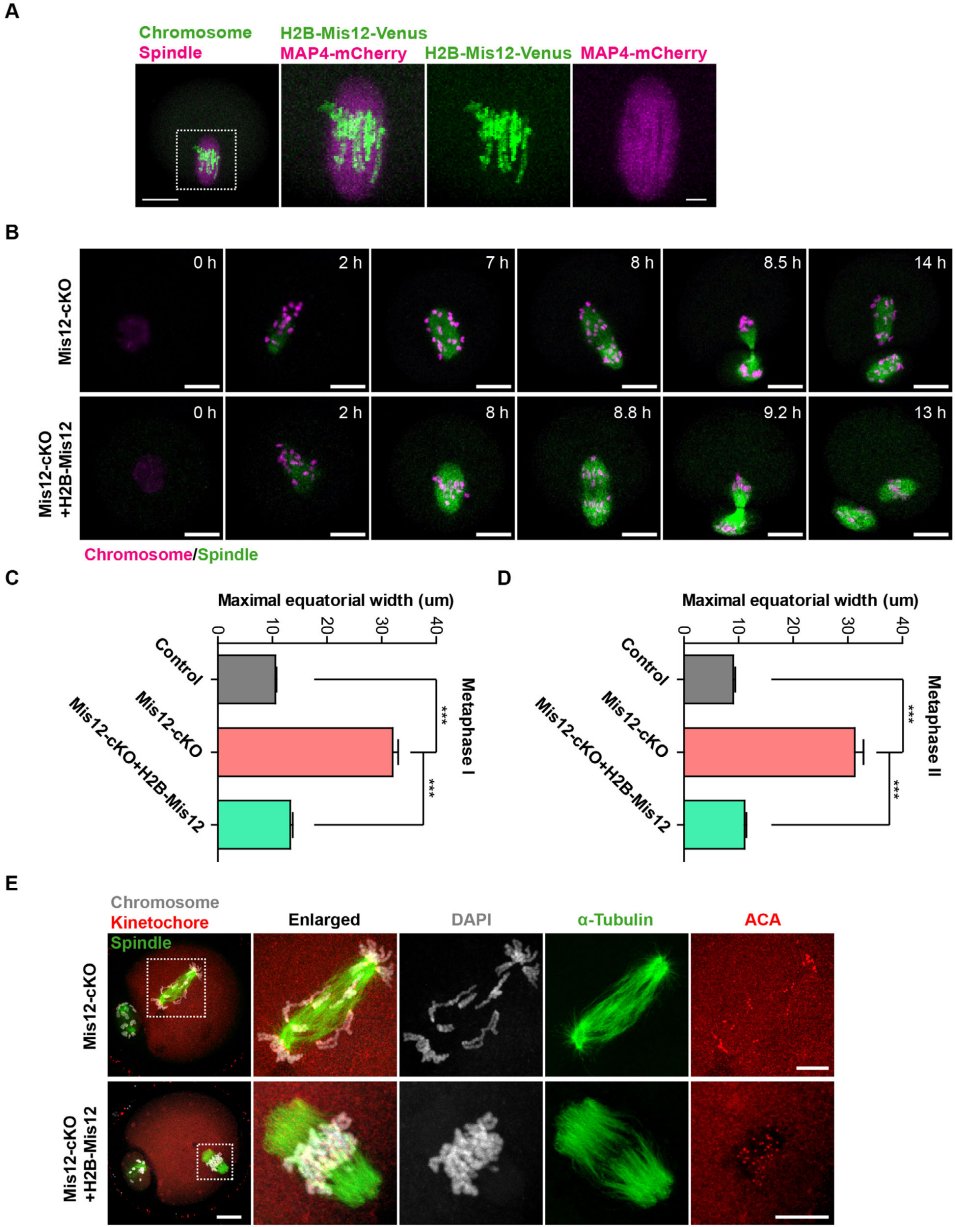
Chromosome‐anchoring of MIS12 rescues K‐MT attachment in *Mis12*‐deleted oocytes. (A) The H2B‐MIS12 fusion protein successfully localizes to chromosomes in mouse oocytes. Microtubules (magenta) and chromosomes (green) were visualized using MAP4‐mCherry and H2B‐MIS12‐Venus in live oocytes, respectively. Scale bars: 20 µm (overview), 5 µm (enlarged view). (B) Representative time‐lapse images of oocytes expressing EGFP‐MAP4 (microtubules) and H2B‐mCherry (chromosomes), comparing *Mis12*‐deleted oocytes with or without supplementation of *H2B*‐*Mis12* fusion mRNA. Images were captured over 13 h with a frame interval of 10 min. Scale bar: 20 µm. Three independent experiments were conducted. (C) Chromosome alignment in meiosis I is restored by expressing the H2B‐MIS12 fusion protein in *Mis12*‐deleted oocytes (Control, n = 16; *Mis12*‐cKO, n = 18; *Mis12*‐cKO + *H2B*‐*Mis12*, n = 17). Data are presented as mean ± SEM; ^***^ *p* < 0.0001. (D) Chromosome alignment in meiosis II is restored by expressing the H2B‐MIS12 fusion protein in *Mis12*‐deleted oocytes (Control, n = 15; *Mis12*‐cKO, n = 13; *Mis12*‐cKO + *H2B*‐*Mis12*, n = 16). Data are presented as mean ± SEM; ^***^ *p* < 0.0001. (E) K‐MT attachment in meiosis II is rescued by H2B‐MIS12 (*Mis12*‐cKO, n = 12; *Mis12*‐cKO + *H2B*‐*Mis12*, n = 13). Oocytes were immunostained with anti‐α‐tubulin and anti‐ACA antibodies. DNA was counterstained with DAPI. Scale bars: 20 µm (overview), 5 µm (enlarged view).

Given that MIS12 is essential for K‐MT attachment during oocyte meiosis, we reasoned that tethering it directly to chromosomes might restore this function in *Mis12*‐deleted oocytes. To test this possibility, an H2B‐anchored MIS12 fusion probe was constructed and used for the rescue experiment. The localization and expression of H2B‐MIS12‐Venus specifically showed that H2B effectively anchored MIS12 to the chromosomes (Figure 8A). Then, the *H2B‐Mis12* construct was used for microinjection in *Mis12*‐deleted oocytes. As expected, the live imaging result showed that chromosome behavior and alignment in *Mis12*‐deleted oocytes were well rescued by H2B‐MIS12 (Figure 8B and Videos S10 and S11). Chromosome alignment in meiosis I and meiosis II was significantly rescued (Figure 8C,D). The result of kinetochore staining clearly showed that chromosome alignment and K‐MT attachment were rescued in *Mis12*‐deleted oocytes by H2B‐MIS12 (Figure 8E). These results provide strong evidence that MIS12 directly interacts with microtubules and that the MIS12‐TUBB interaction plays a crucial role in K‐MT attachment during oocyte meiosis.

### 2.9 MIS12 Is Required for SAC Recruitment in Oocyte Meiosis

**FIGURE 9:**
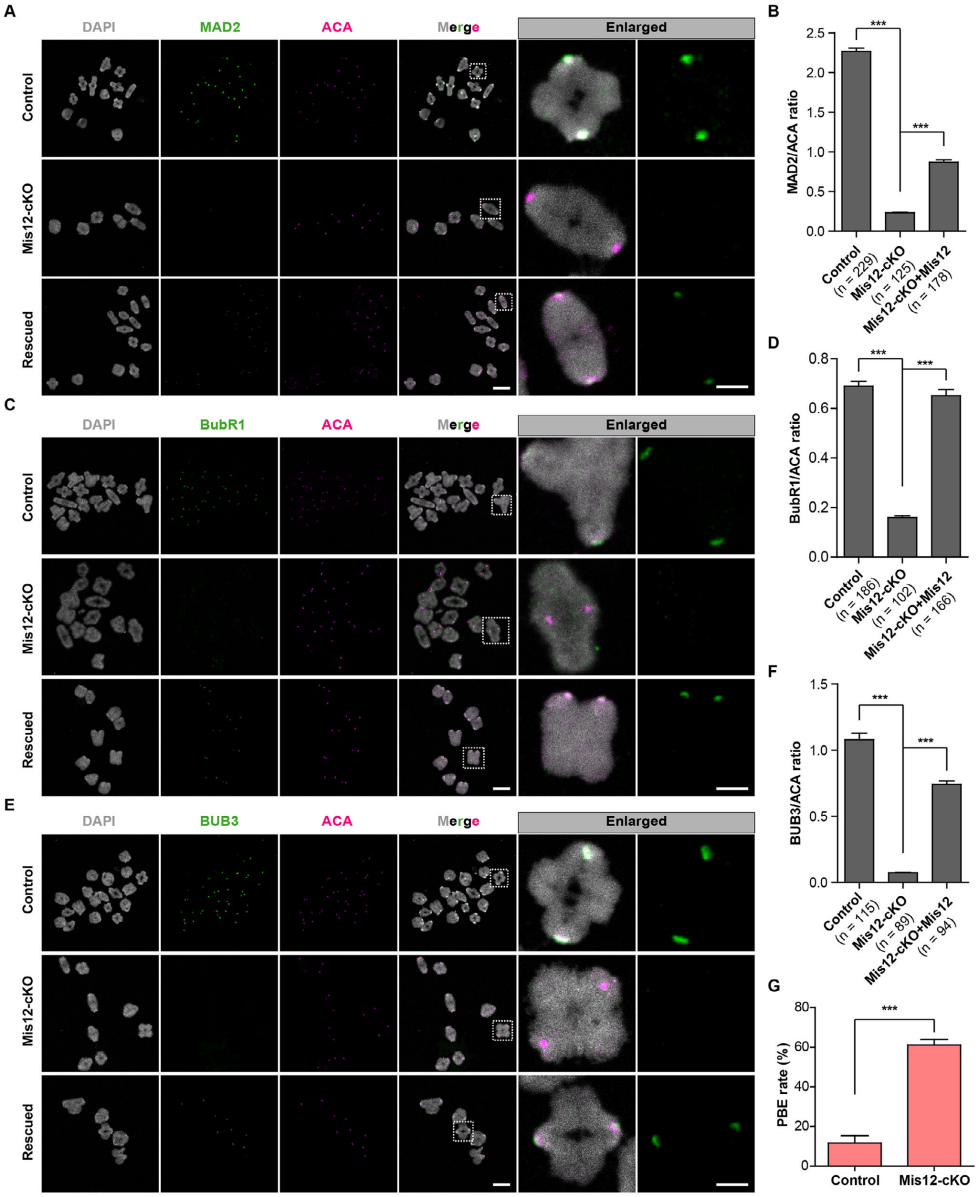
MIS12 is essential for SAC recruitment. (A, C, E) MIS12 is essential for the kinetochore recruitment of SAC components. Representative images of oocytes immunostained for (A) MAD2, (C) BubR1, and (E) BUB3 (all green), together with ACA (magenta). DNA was counterstained with DAPI (grayscale). Scale bars: 10 µm (overview), 5 µm (enlarged view). Applies to A, C, E. Three independent experiments were conducted. (B, D, F) Quantification of the kinetochore fluorescence ratio of (B) MAD2/ACA, (D) BubR1/ACA, and (F) BUB3/ACA in control and *Mis12*‐deleted oocytes from (A), (C), and (E), respectively. Data are presented as mean ± SEM; ^***^ *p* < 0.0001. The number of kinetochores analyzed (n) is indicated for each group. (G) Analysis of PBE rates after nocodazole treatment in control (n = 54) and *Mis12*‐deleted (n = 51) mouse oocytes. Data are presented as mean ± SEM; ^***^ *p* < 0.001.

As shown above, *Mis12*‐deleted oocytes extruded the first polar body prematurely with abnormal separation of the spindle and chromosomes. It was possible that the SAC function was impaired in meiosis I in the *Mis12*‐deleted oocytes. In order to address this issue, we examined the kinetochore localization of three representative SAC proteins: MAD2, BubR1, and BUB3. Immunofluorescence analysis revealed a notable reduction in these three proteins on the kinetochores of bivalents in *Mis12*‐deleted oocytes (Figure 9A–F), suggesting that MIS12 is crucial for recruiting SAC proteins in oocyte meiosis. To validate the requirement of MIS12 for SAC recruitment, rescue experiments were carried out simultaneously with exogenous MIS12. In our expectation, the supplementation of MIS12 effectively increased the expression level of the three SAC proteins (Figure 9A–F). Thus, MIS12 is important and necessary for the recruitment of SAC proteins during meiosis in mouse oocytes. To test the sensitivity of the SAC in the presence or absence of MIS12, we performed a nocodazole treatment experiment in control and *Mis12*‑deleted oocytes. The results showed that the PBE rate was significantly higher in *Mis12*‑deleted oocytes than in control oocytes (Figure 9G), demonstrating that MIS12 is required for proper SAC activation.

### 2.10 MIS12 Is Required for the Stability of KNL1 Assembly

**FIGURE 10:**
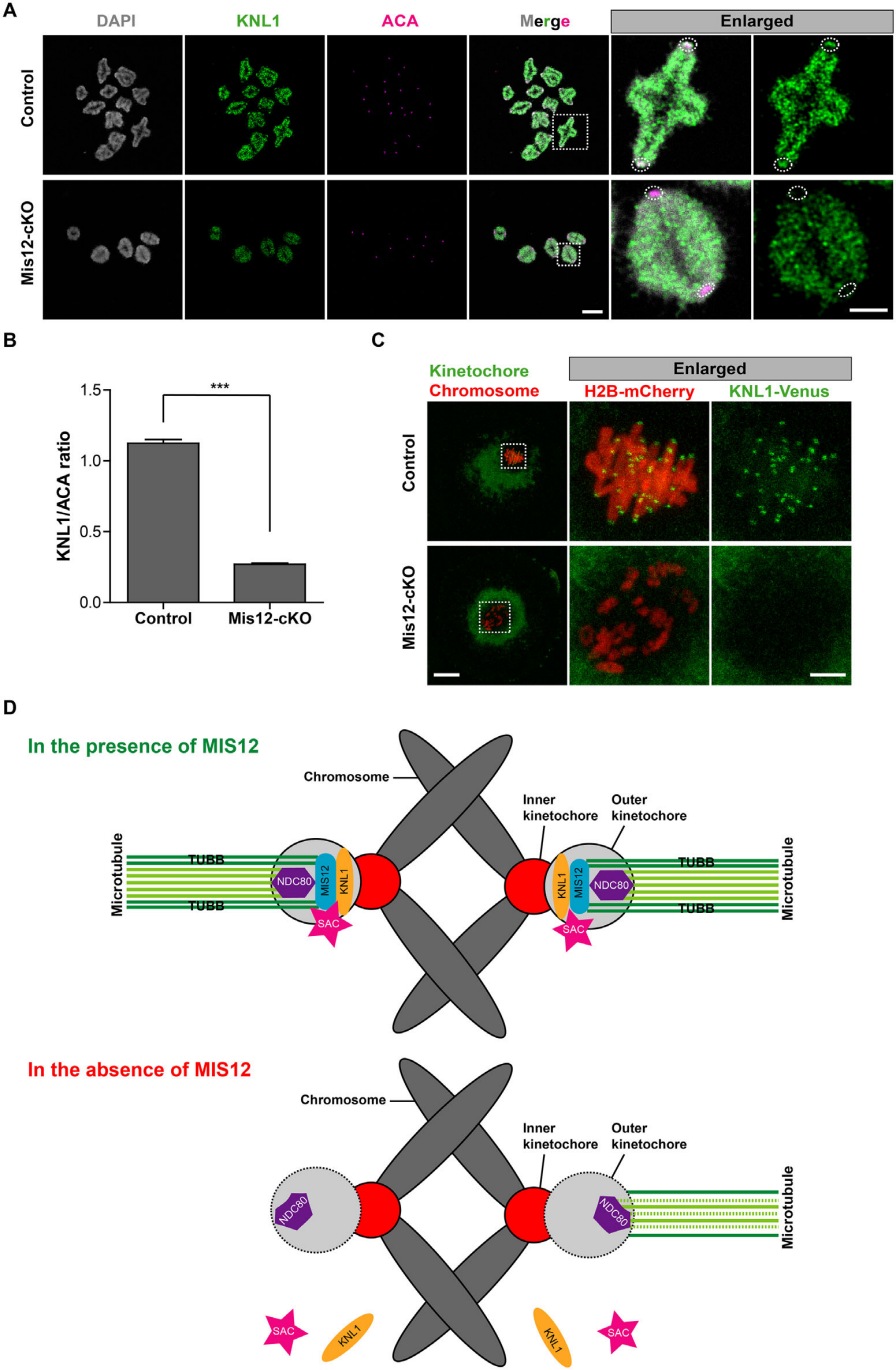
MIS12 is required for KNL1 assembly at kinetochores. (A) KNL1 is depleted from kinetochores in *Mis12*‐deleted oocytes. Oocytes were co‐immunostained with anti‐KNL1 and anti‐ACA antibodies. DNA was counterstained with DAPI. Scale bars: 10 µm (overview), 5 µm (enlarged view). Three independent experiments were conducted. (B) Quantification of relative KNL1 levels at kinetochores in control (n = 253) and *Mis12*‐deleted (n = 152) oocytes, presented as the KNL1/ACA fluorescence intensity ratio. Data are presented as mean ± SEM; ^***^ *p* < 0.0001. The number of kinetochores analyzed (n) is indicated. (C) Exogenous KNL1‐Venus fails to localize to kinetochores in the absence of MIS12 (Control, n = 16; *Mis12*‐cKO, n = 17). Live oocytes expressing KNL1‐Venus (green) and H2B‐mCherry (red) were imaged by confocal microscopy. Scale bars: 20 µm (overview), 5 µm (enlarged view). Three independent experiments were conducted. (D) A model depicting the role of MIS12 in K‐MT attachment during oocyte meiosis. The presence of MIS12 stabilizes bipolar K‐MT attachments through maintaining the function of NDC80 and its interaction with TUBB. This, in turn, promotes KNL1 assembly and subsequent SAC protein recruitment to kinetochores. Conversely, the absence of MIS12 perturbs bipolar K‐MT attachment and abolishes the kinetochore localization of both KNL1 and SAC components.

Taken KNL1 is responsible for the recruitment of components of the SAC. This raises a question of whether the deletion of MIS12 disturbed the SAC function by abolishing the stability of KNL1 assembly. To investigate this hypothesis, we conducted immunofluorescence analysis of KNL1 in control and *Mis12*‐deleted oocytes. The results demonstrated that KNL1 was absent from the kinetochores of *Mis12*‐deleted oocytes but was clearly detected in control oocytes (Figure 10A,B). To further verify the regulation of KNL1 localization by MIS12, an exogenous *Knl1*‐*Venus* fused construct was used for microinjection and examination. As shown in Figure 10C, the KNL1‐Venus was obviously detected on the kinetochores in control oocytes, but it had disappeared in *Mis12*‐deleted oocytes. These findings suggest that the stability of the KNL1 assembly is dependent on MIS12, providing an explanation for the impaired SAC function in *Mis12*‐deleted oocytes. Additionally, the loss of KNL1 localization in the absence of MIS12 indicates that the assembly of the SAC machinery is compromised, leading to potential defects in the proper segregation of chromosomes during meiosis.

## 3 Discussion

Our work fundamentally revises the model of MIS12 function in meiosis by demonstrating its kinetochore localization and NDC80‐independent role in K‐MT attachment—mediated in part by direct TUBB interaction. Beyond this, we uncover a function for MIS12 in stabilizing KNL1 and sustaining SAC signaling, expanding its repertoire from structural scaffold to active regulator of both microtubule binding and checkpoint control (Figure 10D).

The NDC80 complex, a core component of the conserved KMN network, serves as the primary site for K‐MT attachment during mitosis. This role has been further corroborated in meiosis, as evidenced by the severe K‐MT attachment defects observed in *Ndc80*‐deficient oocytes [28]. In Caenorhabditis elegans, KNL1 exhibits a lower microtubule‐binding affinity compared to the NDC80 complex, and its function appears to rely on the presence of the MIS12/MIND complex, which likely potentiates the interaction between NDC80 and microtubules [31, 32]. However, our recent findings reveal that specific ablation of KNL1 in mouse oocytes does not disrupt K‐MT attachment, and the mutant females retain normal fertility (data not shown). In contrast, deletion of MIS12 profoundly impairs K‐MT attachment in mouse oocytes, resulting in complete female infertility. Notably, NDC80 maintains its kinetochore localization in *Mis12*‐deleted oocytes, indicating that the defects in K‐MT attachment are directly caused by the absence of MIS12. The discovery of the MIS12‐TUBB3 interaction, along with the successful rescue of K‐MT attachment through either NDC80‐anchored or H2B‐anchored MIS12 localization in *Mis12*‐deleted oocytes, strongly demonstrates the essential role of MIS12 in K‐MT attachment in mouse oocytes. The distribution of TUBB3 within the meiotic spindle of control and Mis12‐deleted oocytes (Figure S5) suggests that the MIS12‐TUBB3 interaction is vital for establishing K‐MT attachment. Moreover, the detection of an interaction between MIS12 and TUBB5 further reveals that the MIS12–β‐tubulin association is not limited exclusively to TUBB3, indicating that MIS12 recognizes conserved structural motifs within the β‐tubulin family. Functionally, the ability of MIS12 to bind multiple β‐tubulin isoforms (including TUBB3 and TUBB5) underscores its role as a robust and versatile microtubule‐binding component. We initially focused on TUBB3 because it was the primary candidate identified in our yeast two‐hybrid (Y2H) screen. Meanwhile, functional redundancy among TUBB isoforms likely ensures stable K‐MT attachment even when the composition of tubulin isoforms varies. Overall, it is proposed that the synergetic interactions between both NDC80 and MIS12 with microtubules are required for proper K‐MT attachment during oocyte meiosis. This cooperative mechanism likely involves NDC80 providing the core microtubule‐binding activity, while MIS12 contributes to the stabilization or regulation of the attachment through its direct interaction with microtubules, particularly the β‐tubulin isoforms, with their functions being mutually reinforcing. In the unique context of acentrosomal spindle assembly in oocytes, such multifaceted interactions are crucial for enabling kinetochores to efficiently capture and stabilize microtubules emanating from multiple directions, thereby ensuring correct chromosome alignment and segregation. The elucidation of this mechanism not only highlights the specialized configuration of collaborative interactions within the KMN network in female germ cells but also provides fresh molecular insights into the mechanisms ensuring fidelity in mammalian meiotic division.

In *Mis12*‐deleted mouse oocytes, NDC80 retains its kinetochore localization while KNL1 fails to do so. This differential behavior can be attributed to the hierarchical and context‐dependent assembly mechanism of the KMN network. Firstly, the inner kinetochore complex CCAN, particularly CENP‐C, serves as a crucial upstream recruiter for the KMN complex [33]. In mammalian systems, CENP‐C has been demonstrated to directly recruit the MIS12 complex through its C‐terminal region. The MIS12 complex then acts as a central scaffold, simultaneously interacting with and stabilizing both the NDC80 complex and KNL1 [30]. Therefore, the absence of MIS12 directly disrupts this scaffold, leading to the failure of KNL1 recruitment. However, NDC80's retention suggests the existence of a parallel or compensatory recruitment pathway that is independent of MIS12 in mouse oocytes. One strong candidate is the CENP‐T pathway. CENP‐T, another CCAN component, can directly bind to the NDC80 complex and facilitate its kinetochore loading, a pathway that is known to be functionally important in both mitosis and meiosis [3, 29, 33]. It is plausible that in the unique meiotic environment of oocytes, the CENP‐T‐dependent pathway is sufficiently robust to maintain NDC80 at kinetochores—even in the absence of the MIS12 scaffold—by binding multiple NDC80 dimers through its N‐terminal region, whereas KNL1 recruitment is exclusively dependent on the MIS12 complex. Second, the regulatory landscape is further complicated by distinct kinase dependencies. KNL1 recruitment and stability are likely dependent on SAC‐associated kinase activity at the kinetochore [34, 35, 36, 37]. The absence of MIS12 disrupts the entire KMN network, thereby impairing the localization and function of SAC components; this, in turn, initiates a vicious cycle that further undermines KNL1 stability. The recruitment of NDC80, however, is largely governed by cyclin‐dependent kinase (CDK) through the CENP‐T pathway, whereby CDK1‐mediated phosphorylation enhances the CENP‐T‐NDC80 interaction. Given the unique meiotic kinase environment of oocytes, this CDK‐dependent regulation may remain robust even without MIS12, rendering NDC80 localization more resistant to its loss than that of KNL1. Furthermore, the unique functional demands of the oocyte must be considered. The acentrosomal nature of meiotic spindle assembly places exceptional demands on the K‐MT attachment process. A key adaptation to this challenge appears to be the evolution of redundant pathways (CENP‐C/MIS12 and CENP‐T) that prioritize the stabilization of the core microtubule‐binding element, NDC80. This safeguard ensures a critical last line of defense for microtubule capture under the precarious conditions of female meiosis.

Protein localization is intrinsically linked to its function. The observed K‐MT attachment defects in *Mis12*‐deleted oocytes align with the expected kinetochore localization of MIS12 in mouse oocytes.

While our cKO model definitively proves that endogenous MIS12 is physiologically required for K‑MT attachment at its native location, the tethering assay was designed to test whether the mere presence of MIS12 at the kinetochore or on chromatin is sufficient to drive microtubule capture. In rescue experiments, tethering MIS12 to NDC80 rescued the K‑MT attachment defects in *Mis12*‑deleted oocytes, strongly demonstrating that kinetochore‑localized MIS12 is necessary for proper K‑MT attachment in mouse oocytes. Moreover, the observation that H2B‑tethered MIS12 also rescued these defects provides strong support that the presence of MIS12 on chromatin is sufficient to drive microtubule capture. However, previous studies have suggested that MIS12 localizes to spindle poles during meiosis in mouse oocytes [26]. Actually, in our experiments, classical immunofluorescence staining of fixed oocytes failed to detect specific MIS12 signals at either kinetochores or spindle poles, which may be attributable to limited antibody specificity. Should future studies confirm such a bipolar localization pattern—with MIS12 present at both kinetochores and spindle poles—it would imply a more complex and multifaceted role for this protein in meiotic chromosome segregation than previously recognized. This could represent a functional adaptation specific to the acentrosomal spindle assembly pathway in mammalian oocytes.

Interestingly, the meiotic resumption (GVBD, germinal vesicle breakdown) was not affected by *Mis12* deletion (Figure S6A), whereas it was compromised in *Mis12*‐knockdown oocytes. This discrepancy in GVBD phenotype between our *Mis12*‑cKO model and the knockdown model may result from several factors. First, a compensatory mechanism between Cyclin B1 and Cyclin B2 in the knockout system could be involved, as demonstrated in our previous study [38]. Consistently, the CDK1 activity was not affected in *Mis12*‐deleted GV oocytes (Figure S6B). Second, differences in mouse strains used in the two studies may also contribute.

The observation that supplementation with *Mis12* mRNA only partially restored the levels of SAC proteins (Figure 9) may be attributed to several factors. First, MIS12 is a core component of the heterotetrameric MIS12 complex, which also includes DSN1, NSL1, and PMF1. Because the stability and kinetochore localization of these subunits are interdependent, exogenous supplementation of MIS12 alone may not fully re‐establish the precise stoichiometry of the entire complex if other subunits remain limiting or destabilized. Second, the timing of mRNA injection and subsequent protein synthesis may not perfectly align with the critical window for SAC recruitment during oocyte maturation. Finally, it is possible that the initial depletion of *Mis12* induces subtle, irreversible alterations in kinetochore architecture or downstream signaling pathways that cannot be fully reversed by late‐stage supplementation. Nevertheless, the significant partial rescue of SAC proteins, together with the improvement in chromosome alignment, provides strong evidence for the specific and pivotal role of MIS12 in regulating the meiotic SAC and spindle integrity.

Strikingly, whole‐exome sequencing (WES) of 65 clinical samples from our reproductive medicine center revealed *Mis12* mutations in over 60% of the infertile women. The pathological mechanism remains unknown, and elucidating the impact of specific mutations on K‐MT attachment and chromosome segregation warrants further investigation.

In conclusion, this study collectively reshapes the functional model of the KMN network in oocyte meiosis, providing a critical foundation for further investigation into female meiosis and related fertility disorders.

## 4 Materials and Methods

### 4.1 Mice

C57BL/6J background *Mis12*‐flox mice were generated using CRISPR‐Cas9 technology, with exon 3 specifically targeted for loxP sites insertion. To conditionally delete the *Mis12* gene in oocytes, *Mis12* ^flox/flox^ mice were crossed with transgenic mice expressing Cre recombinase under the control of the zona pellucida 3 (ZP3) gene promoter to produce *Mis12* ^flox/flox^; ZP3‐Cre mice. All experimental protocols and animal handling procedures were conducted in accordance with the guidelines and procedures approved by the ethics committee of Peking University Shenzhen Hospital and Shenzhen Peking University‐The Hong Kong University of Science and Technology Medical Center.

### 4.2 Fertility Testing

For fertility testing, six to eight week‐old *Mis12* ^flox/flox^ (Control) and *Mis12* ^flox/flox^; ZP3‐Cre (*Mis12*‐cKO) females were separately mated with wildtype C57BL/6J males for at least six months. Litter sizes were assessed weekly.

### 4.3 Oocyte Collection and Manipulation

Six‐ to eight‐ week‐old *Mis12* ^flox/flox^ (Control) and *Mis12* ^flox/flox^; ZP3‐Cre (*Mis12*‐cKO) females were used in the experiments. To collect GV‐stage oocytes, mice were injected intraperitoneally with 10 U pregnant mare serum gonadotrophin (PMSG) 48 h in advance. For microinjection, denuded oocytes were incubated in M2 medium (M7167; Sigma‐Aldrich) containing 200 µm IBMX at 37°C. For in vitro maturation, oocytes were cultured in M2 medium. To collect metaphase II oocytes, mice were sequentially injected intraperitoneally with 10 U PMSG and, 48 h later, 10 U human chorionic gonadotropin (HCG), with oocytes collected from the oviductal ampulla 14–16 h after the hCG injection. Cumulus cells were removed by hyaluronidase (1 mg/ml, Sigma, H3506) treatment. For nocodazole (Selleck) treatment, a concentration of 100 nM was applied to control and *Mis12*‑deleted oocytes after meiotic resumption, which was achieved by three hours of incubation in nocodazole‑free M2 medium.

### 4.4 CDK1 Kinase Activity Assay

The CDK1 kinase activity assay was performed using the CDK1 Assay Kit (79597, BPS Bioscience) according to the manufacturer's instructions. Briefly, 60 denuded, GV‐intact control and *Mis12*‐cKO mouse oocytes were collected and separately incubated in 20 µL of lysis buffer (5 mM DTT, 20 U/ml RNase inhibitor, and 1% NP‐40) on ice for 30 min. A master mixture was prepared containing 6 µL of 5× Kinase Assay Buffer 1, 1 µL of ATP (500 µM), 5 µL of 10× CDK Substrate Peptide 1, and 18 µL of distilled water. Then, 30 µL of this master mixture was added to each well of a 96‐well plate. The lysed oocytes from the control and *Mis12*‐cKO groups were then added to the wells (each group in triplicate) and incubated at 30°C for 45 min. Subsequently, 50 µL of Kinase‐Glo Max reagent was added to each well, and the plate was covered with aluminum foil and incubated at room temperature for 15 min. Finally, luminescence was measured using a microplate reader (Promega). Distilled water was used as a blank control.

### 4.5 Zygote Collection and Culture

Six‐ to eight‐ week‐old *Mis12* ^flox/flox^ (Control) and *Mis12* ^flox/flox^; ZP3‐Cre (*Mis12*‐cKO) females were used in the experiments. Mice received sequential intraperitoneal administration of 10 U PMSG and, 48 h later, 10 U hCG, followed by cohabiting with wild‐type adult males, respectively. The following morning, vaginal plugs were examined 8 h after mating. Zygotes were then collected from the oviducts and transferred to M16 medium for further culture.

### 4.6 Human Oocyte Experiment

Surplus fresh immature human oocytes (n = 10) were collected from patients aged 25–50 years at the Reproductive Center of Peking University Shenzhen Hospital, with written informed consent obtained from all donors. The oocytes were subjected to chromosome spreading followed by immunofluorescence. All experimental protocols were approved by the Institutional Review Board (IRB) of Peking University Shenzhen Hospital.

### 4.7 Immunofluorescence

Oocytes were fixed in 4% paraformaldehyde (PFA) containing 0.05% Triton X‐100 for 30 min at room temperature (RT), followed by permeabilization with 0.5% Triton X‐100 for 20 min at RT. Subsequently, oocytes were blocked in phosphate‐buffered saline (PBS) supplemented with 1% bovine serum albumin (BSA) for 1 h at RT. For spindle immunostaining, oocytes were incubated overnight at 4°C with an anti–α‐tubulin–Alexa Fluor 488 antibody (1:500, 322588, Thermo Fisher Scientific). The following morning, oocytes were washed three times with wash buffer (PBS containing 0.1% Tween‐20 and 0.01% Triton X‐100) and then counterstained with DAPI (1 µg/mL in PBS; Sigma‐Aldrich) for 15 min. Finally, oocytes were mounted on glass slides using antifade mounting medium and imaged using the Leica STELLARIS 5 confocal microscope system. For centromere immunostaining, a primary human anti‐ACA (anti‐centromere antibody) antibody (1:50; 15–234, Antibodies Incorporated) and a secondary antibody conjugated with Alexa Fluor Cy5 (1:400, 709‐175‐149, Jackson ImmunoResearch) were used.

### 4.8 Cold Treatment

MI oocytes were subjected to a 5 min ice treatment to depolymerize non‐kinetochore microtubules, followed by fixation using the conventional immunofluorescence protocol. Microtubules and centromeres were stained with anti–α‐tubulin–Alexa Fluor 488 antibody (1:500) and anti‐ACA antibody (1:50), respectively.

### 4.9 Plasmid Construction, RNA Preparation, and Microinjection

The mouse *Ndc80*, *Nuf2*, *Mis12*, *Knl1*, and *Tubb3* genes were amplified from mouse ovary cDNA and inserted into the pcDNA3.1 or pcDNA3.1‑Venus vector. The following restriction sites were used: Kpn I and Age I for *Ndc80*; Nhe I and Kpn I for *Mis12*; Nhe I and BamH I for *Knl1*; and Nhe I and Kpn I for *Tubb3*. The *Ndc80*‑*Mis12* and *H2B*‑*Mis12* fusions were constructed and cloned into the pcDNA3.1‑Venus vector between the Kpn I and Age I sites, and between the Nhe I and Kpn I sites, respectively. For linearization, the restriction enzyme Xba I was used for all pcDNA3.1 or pcDNA3.1‑Venus constructs.

The *Mis12* gene was also cloned into the pCS2(+)‑Myc vector between the Fse I and Asc I restriction sites. Linearization was performed using Not I.

The EGFP‑MAP4 and H2B‑mCherry constructs were cloned into the pGEMHE vector (purchased from EUROSCARF). The MAP4‑mCherry construct was cloned into the pGEMHE vector between the Hind III and Not I sites. EGFP‑MAP4 was linearized using Nhe I, whereas H2B‑mCherry and MAP4‑mCherry were linearized using Not I. For microinjection, RNAs were transcribed in vitro with a T7 or SP6 mMessage mMachine kit (Invitrogen) and purified with a RNeasy Mini kit (Qiagen). The purified RNAs were subsequently dissolved in nuclease‐free water, stored at −80°C, and used at a concentration of 500 ng/µl unless indicated otherwise. All microinjections were carried out with an Eppendorf operating system.

### 4.10 Time‐Lapse Confocal Live Imaging

Time‐lapse live‐cell imaging was conducted using a Leica STELLARIS 5 confocal microscope system equipped with a CO2 incubator chamber, maintained at 5% CO2 and 37°C. Digital time‐lapse images were acquired with a 20 × 0.75 objective lens, capturing 15 z‐slices spaced at 3 µm intervals. Image acquisition was controlled by the LAS X software. Prior to imaging, injected oocytes were incubated in M2 medium supplemented with IBMX for at least 3 h, followed by washing and subsequent incubation in M2 medium for time‐lapse observation. To monitor the dynamics of EGFP‐MAP4 and H2B‐mCherry fluorescence, images were captured at 10 min intervals.

### 4.11 Yeast Two‐Hybrid Screening

In this yeast two‐hybrid screening to identify protein interactors of MIS12, the Y2HGold (MATα) strain was utilized, with the bait plasmid pGBKT7‐*Mis12* expressing full‐length *Mis12* and a prey cDNA library constructed from mouse ovarian tissue in the pGADT7 vector. Initially, bait autoactivation and toxicity were evaluated by co‐transforming pGBKT7‐*Mis12* with an empty pGADT7 vector into yeast cells, followed by plating on synthetic defined media lacking tryptophan and leucine (DDO) or lacking tryptophan, leucine, histidine, and adenine (QDO), using pGBKT7‐53 + pADT7‐T as a positive control and pGBKT7‐lam + pGADT7‐T as a negative control. For library screening, cells were co‐transformed with the bait plasmid and the mouse ovarian cDNA library, plated on QDO medium supplemented with 3‐aminotriazole (3‐AT) and X‐α‐gal, and incubated at 30°C for 3–5 days. Positive colonies exhibiting blue coloration were re‐streaked for secondary screening, and putative interacting proteins were identified through colony PCR and sequencing, with results annotated against the NCBI database. A total of 57 clones were ultimately obtained.

### 4.12 Co‐Immunoprecipitation (Co‐IP)

*Myc*‐*Mis12* and *Tubb3*‐*Venus* plasmids were transfected into 293T cells using Lipofectamine 2000 (11668‐019, Invitrogen) according to the manufacturer's instructions. As a control, each plasmid was also transfected individually. After 24–48 h, the cells were lysed in RIPA buffer (50 mM Tris‐HCl, pH 7.5, 150 mM NaCl, 0.1% SDS, 0.5% sodium deoxycholate, and 1% NP‐40) supplemented with protease and phosphatase inhibitors (Sigma) and incubated on ice for 30 min. The lysates were then centrifuged at 14 000 × g for 10 min at 4 °C. The supernatant was collected, and 100 µL of it was incubated with 2 µg of primary antibody overnight with rotation at 4 °C. The next morning, 20 µL of Magnetic Beads Protein A/G (B23201, Selleck) was added, followed by another 4–6 h of incubation with rotation at 4 °C. After washing at least five times with IP lysis buffer (150 mM NaCl, 50 mM Tris‐HCl, pH 7.4, 1 mM EDTA, 0.1% SDS, 1% NP‐40, 0.5% sodium deoxycholate, 0.5 mM DTT, and 1 mM PMSF/protease inhibitor cocktail), the immunocomplexes were eluted with 40 µL of 1× SDS sample buffer and subjected to western blot analysis. Western blotting was performed as previously described (Li et al., 2018). The primary antibodies used were anti‐GFP (1:4000, ab290, Abcam) and anti‐c‐MYC (1:2000, M4439, Sigma‐Aldrich).

### 4.13 Proximity Ligation Assay (PLA)

Protein‐protein interaction between MIS12 and TUBB3 (or TUBB5) was assessed in mouse metaphase I oocytes using the Duolink in situ Proximity Ligation Assay (DUO92101, Sigma‐Aldrich) according to the manufacturer's instructions. Briefly, oocytes were fixed, permeabilized, and incubated with a mixture of primary antibodies, including a rabbit polyclonal antibody against MIS12 and a mouse monoclonal antibody against TUBB3 (or TUBB5). To confirm the specificity of the PLA signal, control experiments were performed in which oocytes were incubated with only the rabbit anti‐MIS12 antibody or only the mouse anti‐TUBB3 (or anti‐TUBB5) antibody. Following primary antibody incubation, the samples were treated with species‐specific PLA probes (anti‐rabbit PLUS and anti‐mouse MINUS), a ligation solution, and an amplification solution. The resulting fluorescent spots, each representing a single protein interaction event, were visualized and acquired by confocal microscopy.

### 4.14 Chromosome Spreads

Oocytes were first treated with Acid Tyrode's solution (Sigma‐Aldrich) at room temperature to completely remove the zona pellucida, while carefully avoiding over‐digestion. After a brief recovery in pre‐warmed M2 medium, the oocytes were transferred onto clean glass slides and fixed in a solution of 1% paraformaldehyde in distilled water (pH 9.2) containing 0.15% Triton X‐100 and 3 mM dithiothreitol, as previously described (Hodges and Hunt, 2002). The slides were placed in a partially sealed humidified chamber and allowed to dry slowly for several hours. Subsequently, the fixed oocytes were blocked with 2% bovine serum albumin (BSA) in PBS for 1 h at room temperature. This was followed by an overnight incubation with primary antibodies at 4 °C. After three washes (10 min each) in wash buffer (PBS containing 0.1% Tween‐20 and 0.01% Triton X‐100), the samples were incubated with appropriate secondary antibodies for 2 h at room temperature.

The following primary antibodies were used: rabbit anti‐NDC80 antibody (1:50, GTX70268, GeneTex), mouse anti‐MYC antibody (1:50, M4439, Sigma‐Aldrich), rabbit anti‐MIS12 antibody (1:50, ab70843, Abcam), rabbit anti‐MIS12 antibody (1:50, bs‐7713R, Bioss), rabbit anti‐MAD2 antibody (1:50, A16395, Abclonal), rabbit anti‐BubR1 antibody (1:50, 11504‐2‐AP, Proteintech), rabbit anti‐BUB3 antibody (1:50, sc‐376506, Santa Cruz), rabbit anti‐KNL1 antibody (1:50, PA5‐114943, Invitrogen) and a human anti‐centromere antibody (ACA; 1:50 dilution; 15–234, Antibodies Incorporated).

The second antibodies are Alexa Fluor 488 goat anti‐mouse IgG (H + L) (A11029); goat anti‐rabbit IgG (H + L) (A11034); Cy5‐conjugated anti‐human secondary antibody (709‐175‐149, Jackson ImmunoResearch).

### 4.15 Statistical and Image Analysis

Statistical analysis was performed using Student's *t*‐test in GraphPad Prism 5. Image analysis was conducted with LAS X (Leica Microsystems) and Adobe Photoshop CS5, and figures were assembled using Adobe Illustrator CC5.

## Author Contributions

Conceptualization: **Jian Li**; Formal analysis: Chun‐Hui Zhang and **Xi Xia**; Investigation: Jian Li, **Cheng‐Yuan Li**, and **Da‐Rong Wen**; Resources: **Liang Zhou** and **Yong Wang**; Writing−original draft: Jian Li; Writing−review & editing: Qing‐Yuan Sun; Supervision: Qing‐Yuan Sun and **Chang‐Zhong Li**; Project administration: Jian Li; Funding acquisition: Jian Li and Wei‐Ping Qian.

## Funding

This work was supported by grants from the National Natural Science Foundation of China (82271684), the Guangdong Basic and Applied Basic Research Foundation (2023A1515010367, 2024A1515030140), the project of Shenzhen Science and Technology Program (JCYJ20220818102809021, JCYJ20250604183726035), and the Shenzhen High‐level Hospital Construction Fund. This work was also supported by Sanming Project of Medicine in Shenzhen (Grant No. SZSM202211043).

## Ethics statement

All experimental protocols and animal handling procedures were conducted in accordance with the guidelines and procedures approved by the ethics committee of Peking University Shenzhen Hospital and Shenzhen Peking University‐The Hong Kong University of Science and Technology Medical Center. All experimental protocols involving human oocytes were approved by the Institutional Review Board (IRB) of Peking University Shenzhen Hospital.

## Conflicts of Interest

The authors declare no conflicts of interest.

## Supporting information




**Supporting File 1**: advs76171‐sup‐0001‐SuppMat.docx.

**Supporting File 2**: advs76171‐sup‐0002‐VideoS1.mov.

**Supporting File 3**: advs76171‐sup‐0003‐VideoS2.mov.

**Supporting File 4**: advs76171‐sup‐0004‐VideoS3.mov.

**Supporting File 5**: advs76171‐sup‐0005‐VideoS4.mov.

**Supporting File 6**: advs76171‐sup‐0006‐VideoS5.mov.

**Supporting File 7**: advs76171‐sup‐0007‐VideoS6.mov.

**Supporting File 8**: advs76171‐sup‐0008‐VideoS7.mov

**Supporting File 9**: advs76171‐sup‐0009‐VideoS8.mov.

**Supporting File 10**: advs76171‐sup‐0010‐VideoS9.mov.

**Supporting File 11**: advs76171‐sup‐0011‐VideoS10.mov.

**Supporting File 12**: advs76171‐sup‐0012‐VideoS11.mov.

## Data Availability

The data that support the findings of this study are available on request from the corresponding author. The data are not publicly available due to privacy or ethical restrictions.
