# Supplementary material for: MIS12 Is Required for Kinetochore‐Microtubule Attachment in Oocyte Meiosis
Source: Adv Sci (Weinh). 2026 Jun 24:e76171. Online ahead of print. doi: 10.1002/advs.76171 (PMC13336412; doi:10.1002/advs.76171)
Supplement: Supplementary file 1 — Supporting File 1: advs76171‐sup‐0001‐SuppMat.docx. [file ADVS-9999-e76171-s001.docx]

**Supplementary Figures**


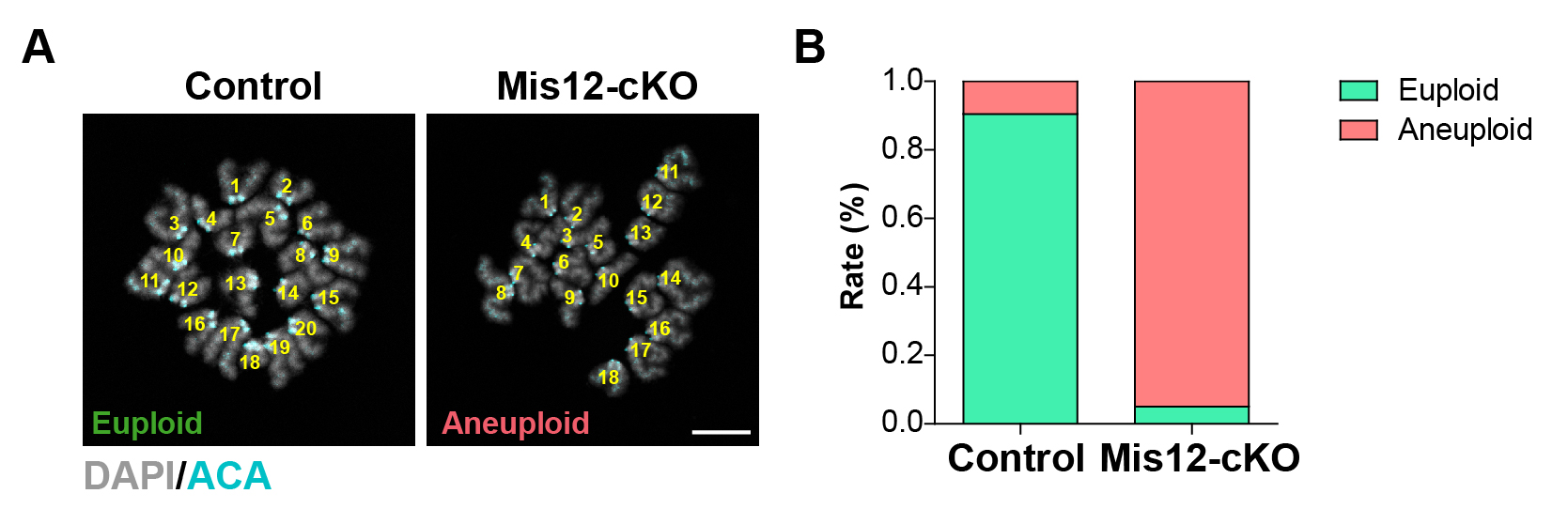


**Figure S1. *Mis12* deletion results in severe aneuploidy in mouse oocytes.**

(A) Chromosome spreading analysis of control and *Mis12*‑deleted MII oocytes. Kinetochores were immunostained with anti‑ACA antibodies, and DNA was counterstained with DAPI. Scale bar: 10 μm.

(B) Statistical analysis of aneuploidy rates in control (n = 21) and Mis12‑deleted (n = 20) oocytes.


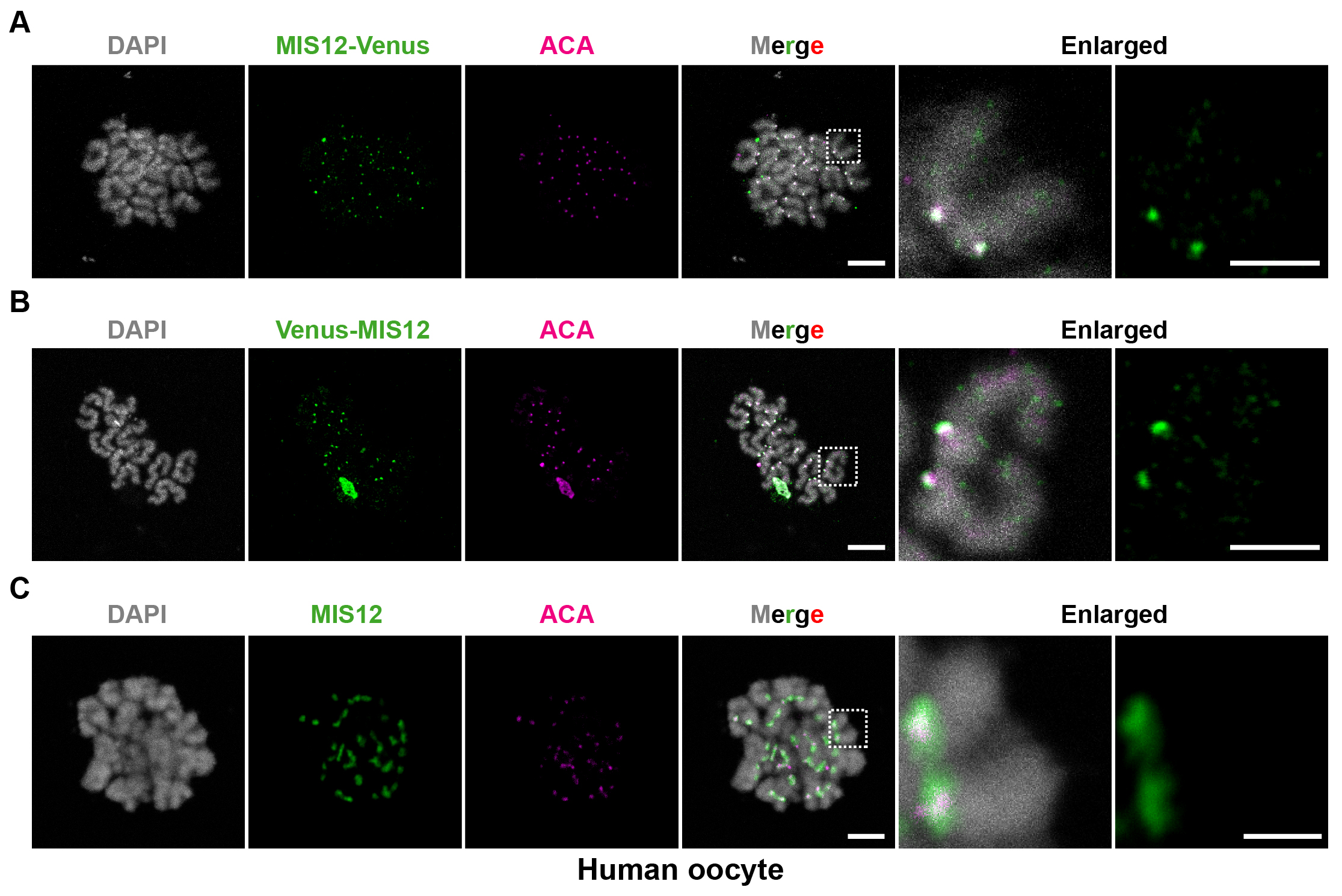


**Figure S2. MIS12 localizes at the kinetochores in mouse and human oocytes.**

(A) MIS12-Venus localized at the kinetochores in mouse oocytes.

(B) Venus-MIS12 localized at the kinetochores in mouse oocytes. Oocytes in (A) and (B) were co-immunostained with anti-GFP and anti-ACA antibodies. Scale bar: 10 μm (overview) and 5 μm (enlarged view).

(C) MIS12 localized at the kinetochores in human oocytes. Oocytes were co-immunostained with anti-MIS12 and anti-ACA antibodies. DNA was counterstained with DAPI. Scale bar: 5 μm (overview) and 2 μm (enlarged view).


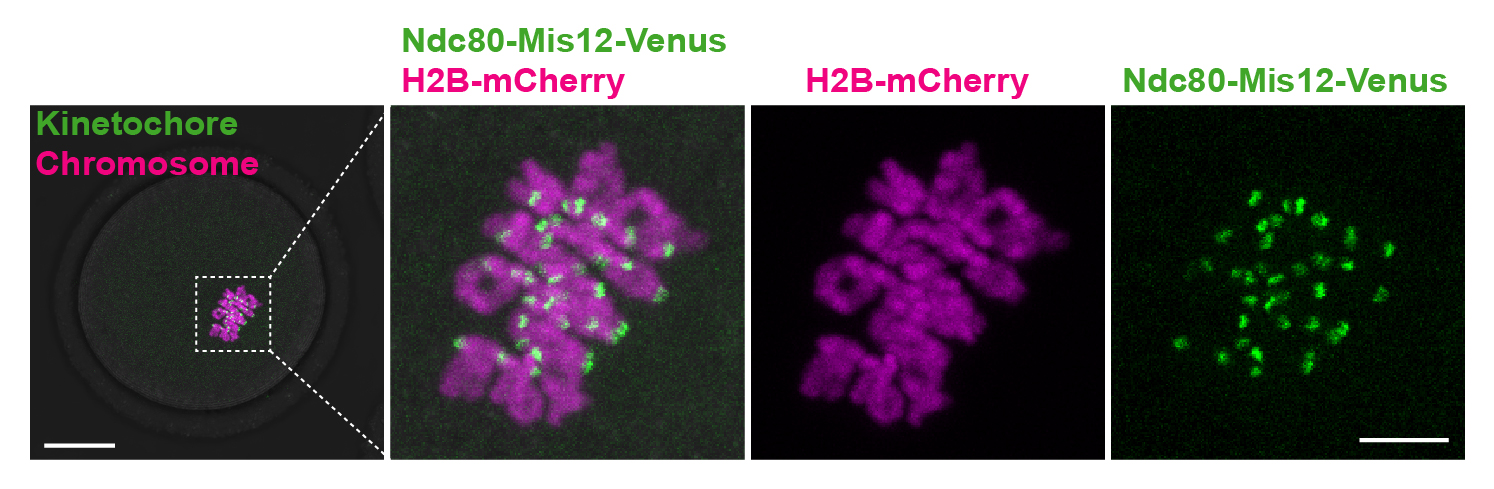


**Figure S3. NDC80 successfully anchors MIS12 on the kinetochores in mouse oocytes.** Live oocytes microinjected with mRNA encoding NDC80-MIS12-Venus (green) and H2B-mCherry (magenta) were imaged by confocal microscopy. Scale bars: 20 μm (overview) and 5 μm (enlarged view).


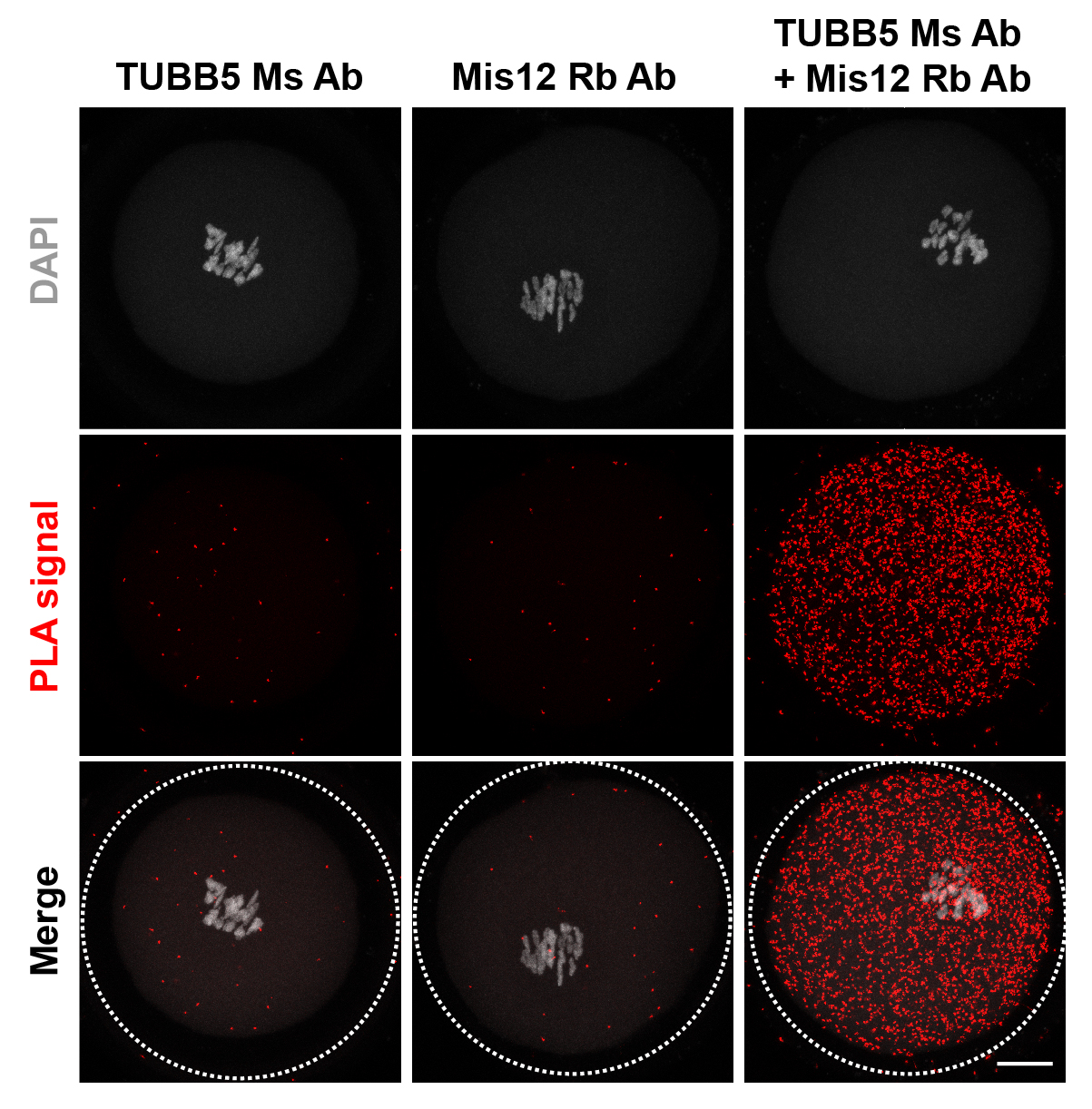


**Figure S4. Proximity ligation assay (PLA) detecting endogenous MIS12-TUBB5 interaction in oocytes.** Wild-type oocytes were co-incubated with anti-MIS12 (rabbit polyclonal) and anti-TUBB5 (mouse monoclonal) antibodies (n = 21). Oocytes incubated with anti-TUBB5 antibody (n = 19) or anti-MIS12 antibody (n = 18) served as negative controls. DNA was counterstained with DAPI. Scale bar: 20 μm.


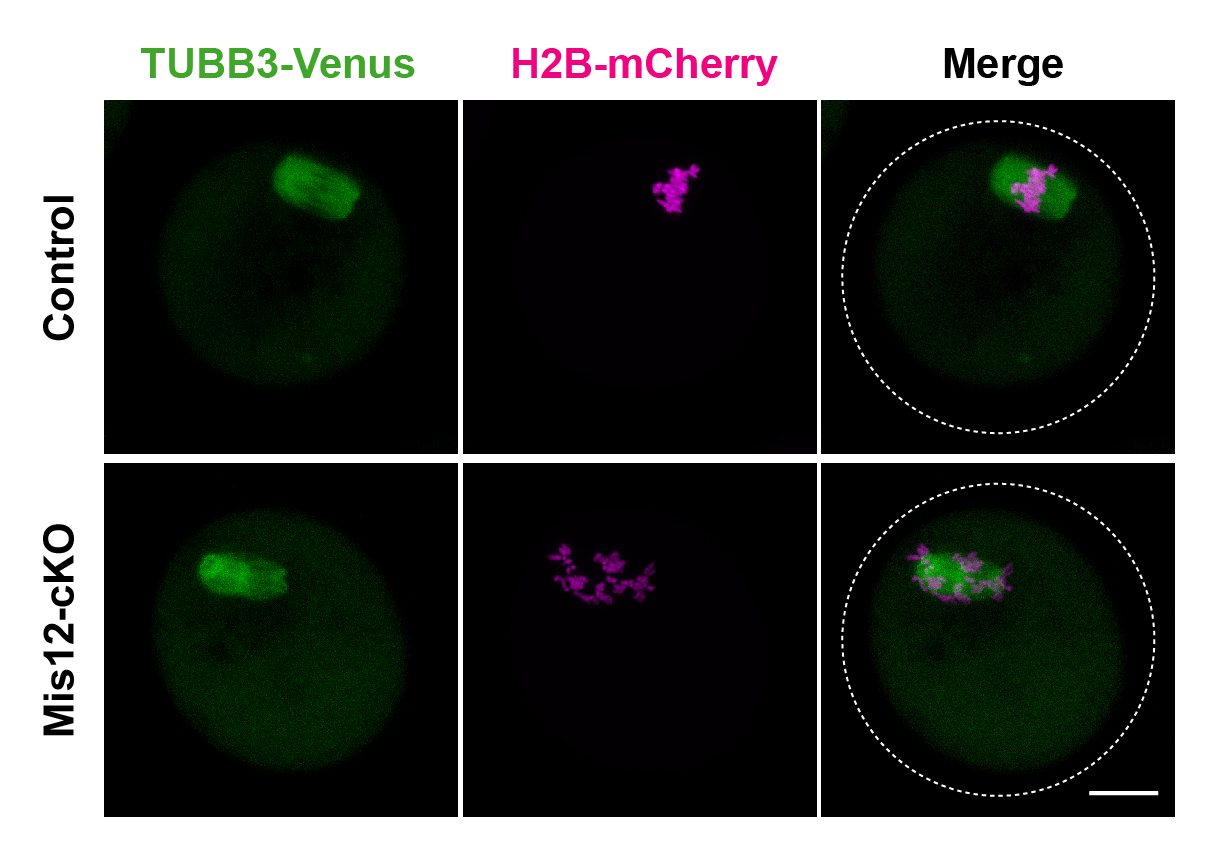


**Figure S5. Analysis of TUBB3 distribution in control (n = 26) and *Mis12*‑deleted (n = 28) oocytes.** *Tubb3*‑*Venus* and *H2B*‑*mCherry* mRNAs were microinjected into control and *Mis12*‑deleted GV oocytes. The oocytes were then incubated in M2 medium containing IBMX for three hours. Confocal imaging and analysis were performed six hours after release into IBMX‑free M2 medium. Scale bar: 20 μm.


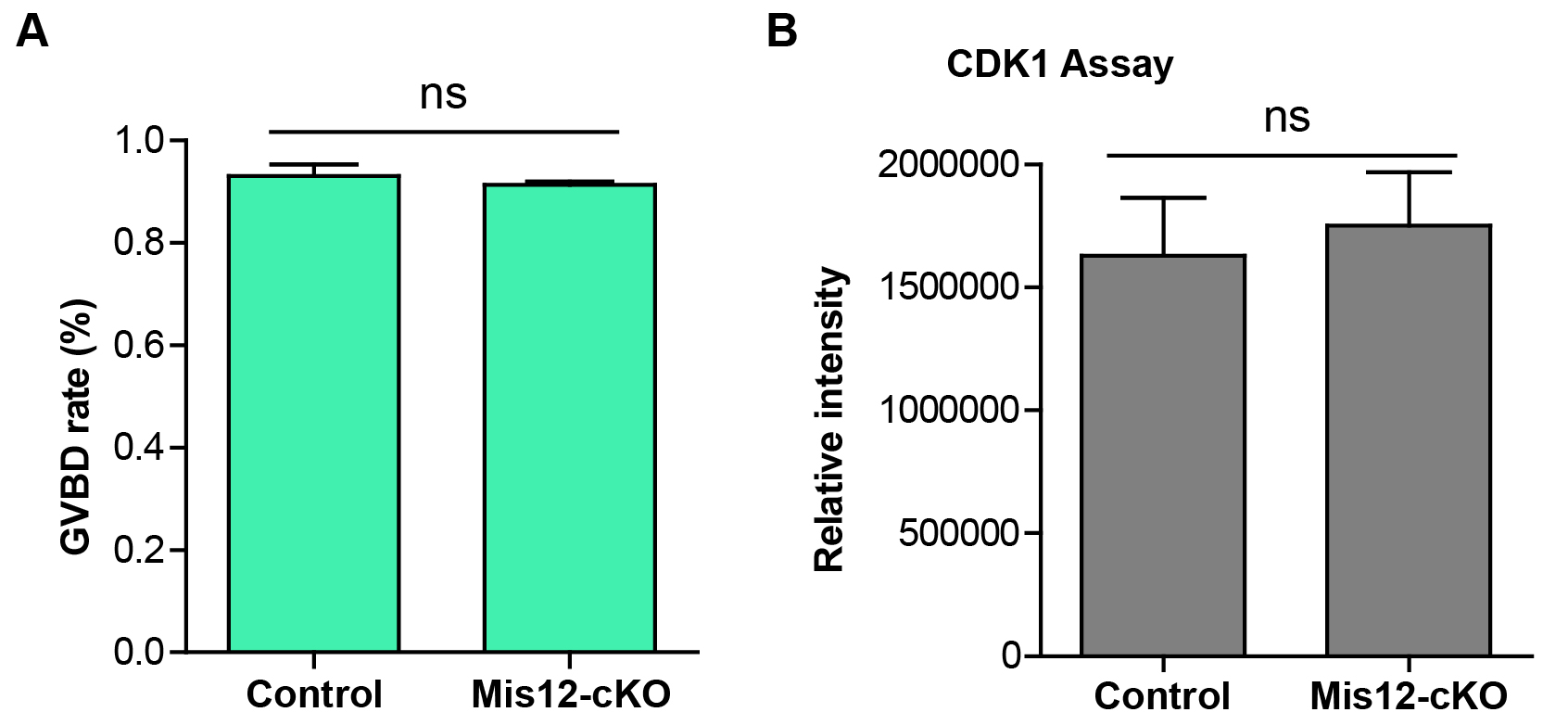


**Figure S6. Meiotic resumption proceeds normally in *Mis12*‑deleted oocytes.**

(A) Analysis of GVBD rates after three hours of incubation in control (n = 171) and *Mis12*‑deleted (n = 127) oocytes. Data are presented as mean ± SEM; ns, not significant.

(B) Analysis of CDK1 activity in GV oocytes from control and *Mis12*‑deleted oocytes. Data are presented as mean ± SEM; ns, not significant.

**Supplementary movie legends**

Movie 1. Chromosome and spindle dynamics during meiotic progression in a control oocyte (corresponds to data in Figure 2A).

Movie 2. Chromosome and spindle dynamics during meiotic progression in a *Mis12*-deleted oocyte (corresponds to data in Figure 2A).

Movie 3. Chromosome and spindle dynamics during meiotic progression in a *Mis12*-deleted oocyte (corresponds to data in Figure 3D).

Movie 4. Chromosome and spindle dynamics during meiotic progression in a *Mis12*-deleted oocyte rescued with *Ndc80* mRNA (corresponds to data in Figure 3D).

Movie 5. Chromosome and spindle dynamics during meiotic progression in a control oocyte (corresponds to data in Figure 4D).

Movie 6. Chromosome and spindle dynamics during meiotic progression in a *Mis12*-deleted oocyte rescued with *Ndc80* mRNA (corresponds to data in Figure 4E).

Movie 7. Chromosome and spindle dynamics during meiotic progression in a *Mis12*-deleted oocyte (corresponds to data in Figure 6A).

Movie 8. Chromosome and spindle dynamics during meiotic progression in a *Mis12*-deleted oocyte rescued with *Mis12* mRNA (corresponds to data in Figure 6A).

Movie 9. Chromosome and spindle dynamics during meiotic progression in a *Mis12*-deleted oocyte rescued with *Ndc80*-*Mis12* mRNA (corresponds to data in Figure 6A).

Movie 10. Chromosome and spindle dynamics during meiotic progression in a *Mis12*-deleted oocyte (corresponds to data in Figure 8B).

Movie 11. Chromosome and spindle dynamics during meiotic progression in a *Mis12*-deleted oocyte rescued with *H2B*-*Mis12* mRNA (corresponds to data in Figure 8B).
